# Total Synthesis of Isoriccardin C and Isoriccardin D Based on a Hydroxyl-Directed Palladium-Catalyzed Intramolecular C–H Alkenylation

**DOI:** 10.1021/acs.orglett.6c00911

**Published:** 2026-04-06

**Authors:** Pablo Losada, José Luis Mascareñas, Moisés Gulías

**Affiliations:** Centro Singular de Investigación en Química Biolóxica e Materiais Moleculares (CiQUS) and Departamento de Química Orgánica, 16780 Universidade de Santiago de Compostela, 15782 Santiago de Compostela, Spain

## Abstract

A concise, nine-step total synthesis of isoriccardin C and isoriccardin D has been developed. The strategy centers on the sequential installation of the four aromatic rings of the backbone by using three key transformations: Suzuki coupling, Wittig olefination, and Ullmann coupling. The pivotal step is a palladium­(II)-catalyzed, intramolecular *ortho*-alkenylation that forges the 18-membered macrocyclic core. This streamlined route enables the total synthesis with minimal reliance on protecting groups, and its modular nature offers a versatile platform for the construction of structural analogues.

## Graphical abstract

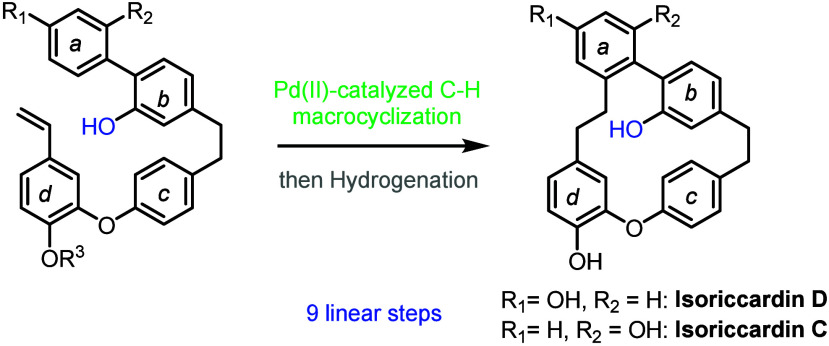

Isoriccardins are a family of cyclic bis­(bibenzyl) natural products, known for their unique cyclic structure featuring biphenyl backbones that can exhibit axial chirality.^1^ They are found in certain plants, especially in liverworts (such as *Marchantia polymorpha*) and possibly other bryophytes or lower plants.^2^ This family of compounds is also closely related to the riccardin and plagiochin groups, which present a different topological C–C bond connection in the biphenyl unit.^3^ All of these compounds, in addition to their intriguing structures, have gained increased attention because of their relevant biological properties. A remarkable example is isoriccardin C, which exhibits antimicrobial (antibacterial and antifungal) properties as well as potential antitumoral/cytotoxic activity. Its related analogue, isoriccardin D, also shows antifungal properties.^4,5^

The syntheses of macrocycles related to isoriccardins C and D have received considerably less attention compared to those of other members of this natural product family, such as riccardin C.^6^ In the case of isoriccardin C, one of the two total syntheses reported employs a Wittig reaction for the key macrocyclization step, in a route requiring up to ∼12 steps, while the other relies on a C–H activation/alkene addition step to accomplish the macrocyclization.^7,8^ The latter is very elegant but requires the installation of a chiral sulfoxide auxiliary to facilitate the C–H activation, which needs to be later removed at an extremely low temperature to avoid racemization. This further increases the overall length up to 16 transformations. With regard to isoriccardin D, the only reported synthesis entails 14 steps and relies on a Wittig reaction for the macrocyclization.^8^

Overall, all the above routes toward isoriccardins are rather linear, are scarcely versatile, and require many protection–deprotection steps. Therefore, the development of more concise and versatile strategies based on catalytic transformations—particularly for the key macrocyclization—remains a significant goal.

As part of our research on palladium­(II)-catalyzed C–H functionalization reations,^9^ we recently developed a new methodology for the kinetic resolution of *ortho*-aryl phenols through a C–H olefination reaction using palladium­(II) catalysis and monoprotected amino acid ligands (MPAAs).^10^

Considering the *ortho*-arylphenol structure of isoriccardins, we recognized that using this C–H functionalization technology in an intramolecular fashion might allow a direct and practical assembly of the macrocyclic skeleton, avoiding the need for introducing extra non-native directing groups. Notably, intramolecular versions of C–H functionalization reactions to synthesize large macrocycles remain largely unexplored,^11^ which added further interest to the study.

Therefore, we proposed the retrosynthetic scheme outlined in Figure 1, which relies on building the biaryl units using key C–C and C–O bond-forming processes. The arene units ***a*** and ***b*** would be joined though a Suzuki–Miyaura cross-coupling, while a Wittig reaction would then be used to connect the **b** and **c** rings. Installation of the ***d*** ring would be carried out through an Ullmann coupling, and the 18-membered macrocyclic structure would ultimately be assembled via a key palladium-catalyzed C–H activation/macrocyclization step.^12^

**Figure 1:**
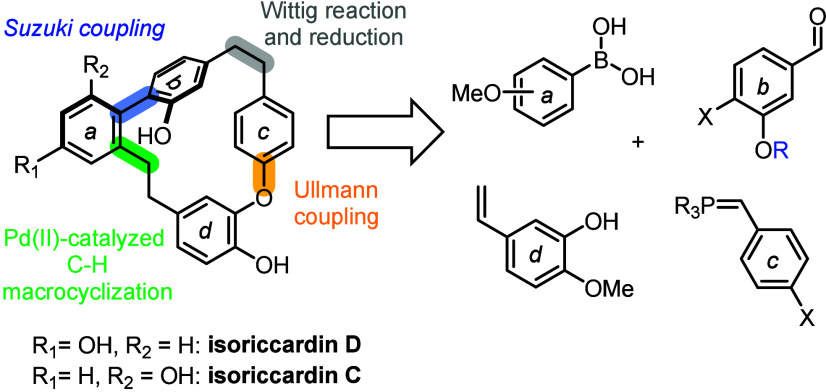
Retrosynthetic overview.

The synthesis started with commercially available 3-hydroxy-4-iodobenzaldehyde (Scheme 1). This compound was protected as the tetrahydropyranyl ether (**1**, 87% yield) and used for a Suzuki coupling with (2-methoxyphenyl)­boronic acid, which is also commercially available. The reaction was efficient, and the coupling product was obtained in an excellent 92% yield. This biaryl product (**2**) was then submitted to a Wittig reaction with commercially available phosphonium salt **S1**, leading to a 1:1.6 *E:Z* mixture in excellent 87% yield. The 18-crown-6 ether was necessary to solubilize the base in dichloromethane. Hydrogenation of the double bond was successfully achieved using in situ-generated diimide from tosylhydrizide and sodium acetate under reflux in THF (**4**, 78% yield). These conditions were chosen since catalytic hydrogenation led to loss of the bromine atom. The next step was the Ullman coupling reaction, which was carried out by treatment of compound **4** with phenol **S2** (prepared in just one step from inexpensive isovanillin; see the Supporting Information) using copper­(I) oxide in pyridine at 140 °C, which led to the corresponding product in 66% yield. Removal of the THP protecting group through mild acid-catalyzed methanolysis at room temperature produced the key cyclization precursor **6** in 91% yield.

**Scheme 1:**
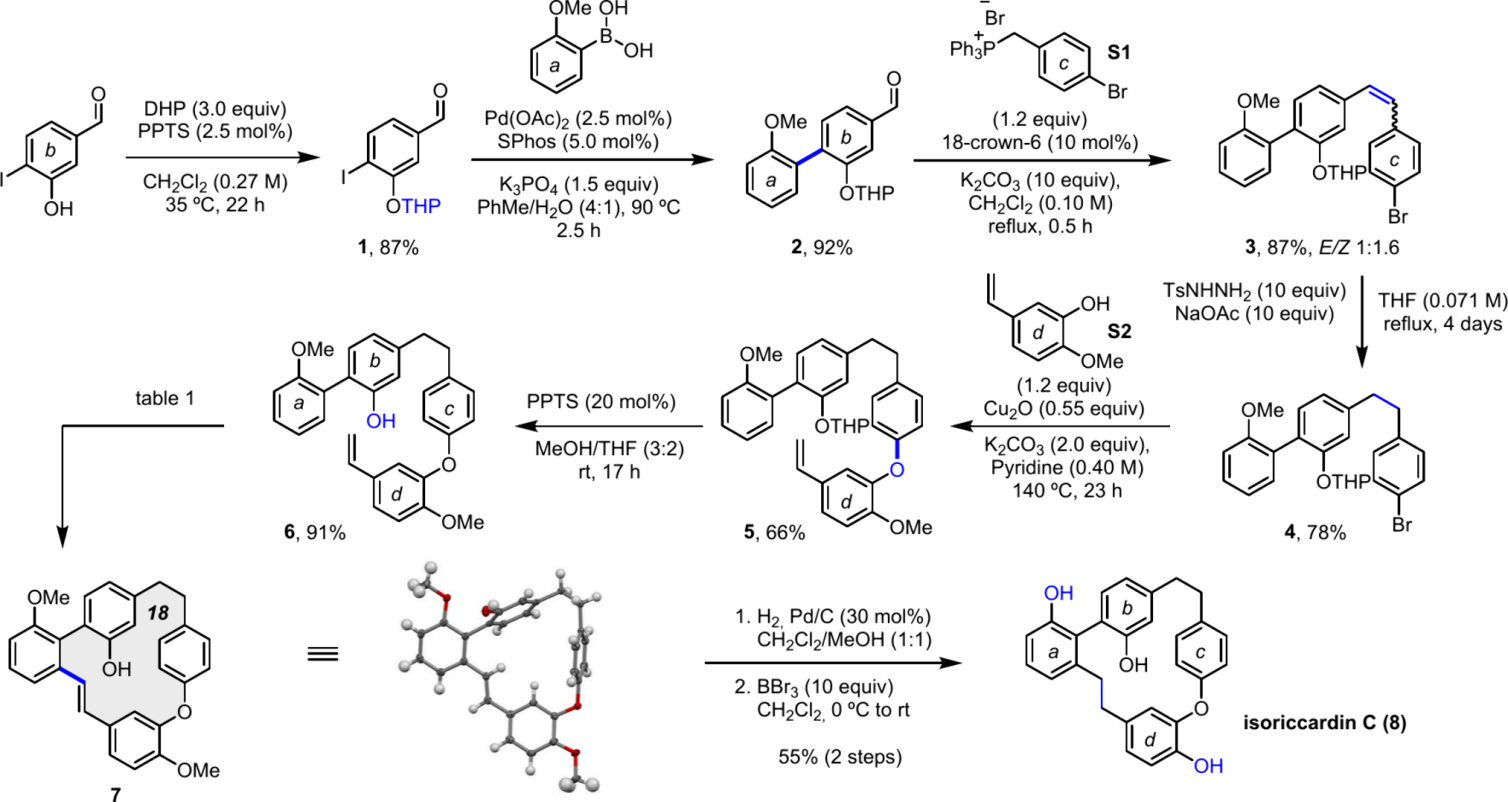
Total Synthesis of Isoriccardin C with a Key Step of Palladium-Catalyzed C–H Macrocyclization

The macrocyclization reaction was initially tested using conditions similar to those optimized for the intermolecular addition of *ortho*-aryl phenols to olefins but under high dilution to avoid intermolecular processes. Using 20 mol% of palladium acetate, Boc-protected leucine, and 1 equiv of copper acetate in *tert*-amyl alcohol under air led to the desired product **7** in 19% yield (after 4 h at 60 °C, Table 1). The structure of this macrocycle was confirmed by X-ray diffraction (CCDC 2526910, Scheme 1).

**Table 1:**
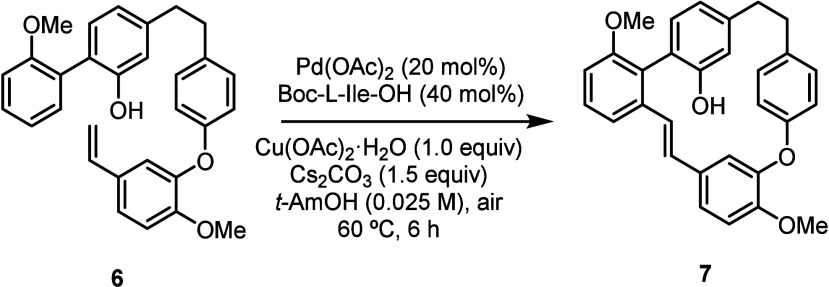
Optimization for the Macrocyclization of **6**

Entry	Deviation from initial conditions	Yield
1	None	19%^b^
2	16 h	–
3	Without Cu^II^, 21 h	11%^c^
4	rt, 26 h	14%^b^
5	0.5 equiv Cu^II^, 45 °C, 17 h	25%^c^
6	Under Ar	30%^b^

a For the general conditions, see Scheme 1.

b Isolated.

c Internal standard.

Longer reaction times (16 h) ensured full conversion; however, the desired product was not detected, suggesting instability of both the product and the starting material under these reaction conditions. Attempts to mitigate decomposition by lowering the temperature slightly improved the results, but the product was still formed in only 14% yield. Assuming that the oxidative environment was responsible for the degradation, we tested the reaction by decreasing the amount of copper salt to 0.5 equiv and conducting it at 45 °C instead of 60 °C; these conditions made it possible to increase the yield up to 25% (determined by internal standard). The optimal results were obtained by employing an argon atmosphere to exclude air, which prevented oxidative degradation. Using 1.0 equiv of copper salt under these conditions provided the product in 30% isolated yield.

With the cyclized product **7** in hand, hydrogenation using Pd/C and a balloon of H_2_ in a 1:1 mixture of dichloromethane and methanol, followed by *O*-demethylation under standard conditions with boron tribromide, gave isoriccardin C (**8**) in 55% yield (combined yield for both steps). Overall, the longest linear synthesis of this route from commercial materials entails 9 steps and an overall yield of 5.4%.

The synthesis of isoriccardin D also started with iodide **1**. Its Suzuki coupling with commercially available (4-methoxyphenyl)­boronic acid gave the expected product in an excellent 85% yield. Subsequent Wittig reaction with phosphonium salt **S1**, followed by reduction with tosylhydrazide, provided compound **10** (74% yield over two steps). Ullmann coupling (73% yield) and deprotection (91% yield), under conditions similar to those previously described for the synthesis of isoriccardin C, led to the acyclic precursor **12**.

After optimization studies, we found that the macrocyclization step proceeded best at 80 °C under an oxygen atmosphere and in the absence of copper salt. After 7 h, the product was obtained in 25% yield with near-complete conversion (94%). This macrocycle was also crystalline, and its X-ray structure is depicted in Scheme 2 (CCDC 2526908). Access to isoriccardin D was achieved via the two-step sequence of hydrogenation and demethylation.

**Scheme 2:**
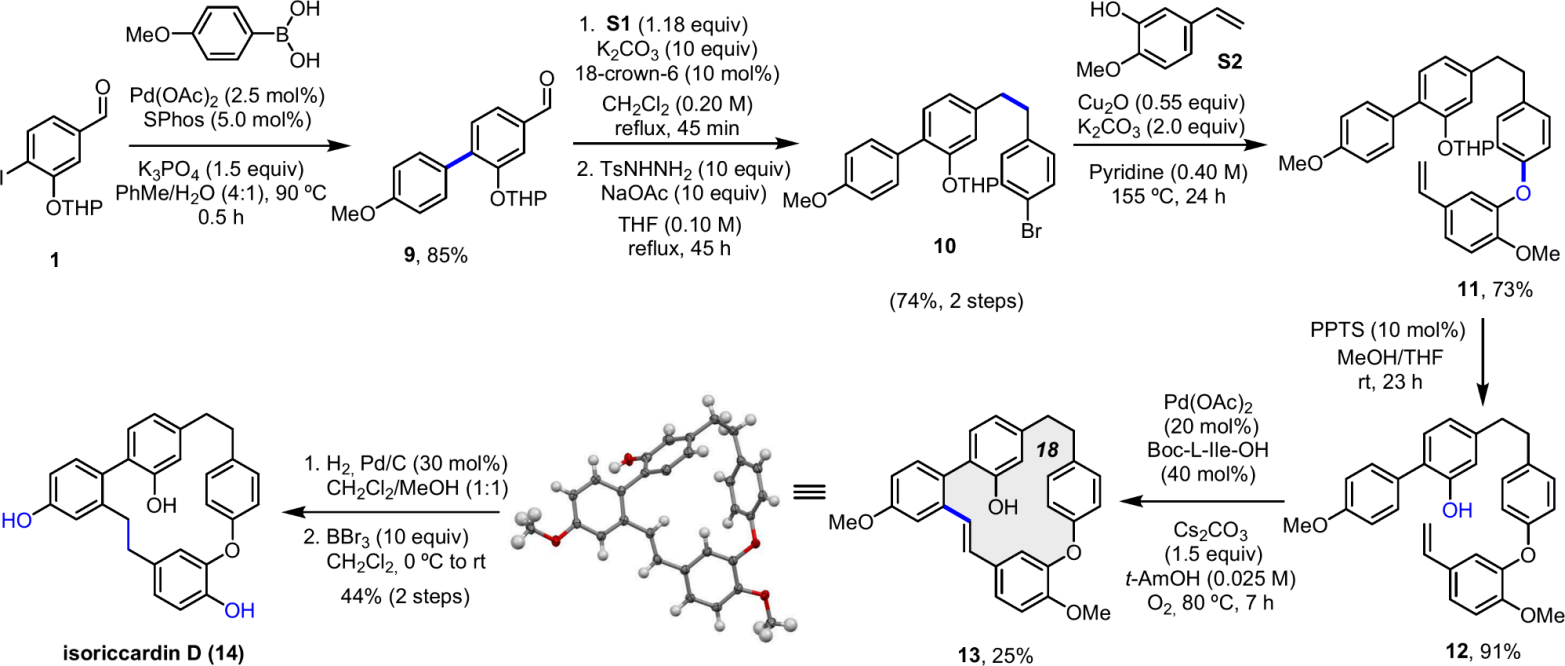
Total Synthesis of Isoriccardin D

The moderate yields in the macrocyclizations are likely because of the sensitivity of these substrates to oxidation and side reactions derived from the electron-rich nature of the arenes. Therefore, we considered the introduction of electron-withdrawing functionalities, such as nitro groups, in conjugation with the olefin to facilitate the intramolecular olefination. The strategy was explored for the synthesis of the nitro derivative of isoriccardin C, shown in Scheme 3. The synthesis started with the biphenyl aldehyde **2**, previously used for the synthesis of isoriccardin C. Wittig reaction with the O-benzyl-protected phosphonium salt **S3** led to the expected alkene in excellent 97% yield. The reduction of the olefin and the cleavage of the O-benzyl group were achieved simultaneously under typical conditions to give the phenol **15**. This compound can then engage in a nucleophilic aromatic substitution with the 3-fluoro-4-nitrostyrene (**S4**; its synthesis is described in the Supporting Information) to give the expected ether **16** in 71% yield, which was easily transformed into the deprotected derivative **17** in 97% yield. After a concise optimization, the macrocyclization reaction was achieved using a reduced amount of anhydrous copper acetate (20 mol%) under an oxygen atmosphere, allowing compound **18** (CDCC 2526903) to be obtained in 57% yield, better than than the yield observed in the previous cyclizations to give the parent products.

**Scheme 3:**
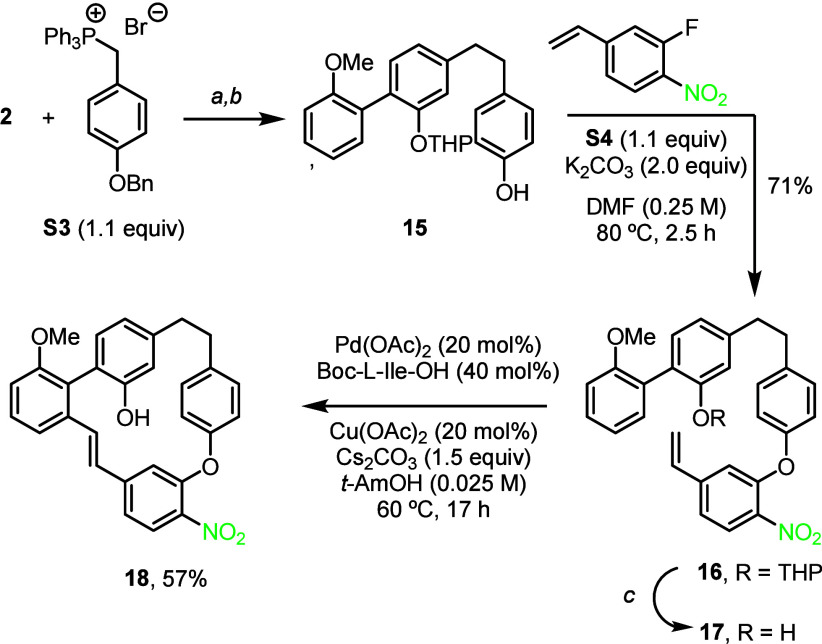
Synthesis of the Nitro Analogue of Isoriccardin C^a^

a Conditions: a) K_2_CO_3_ (10 equiv), 18-crown-6 (10 mol%), CH_2_Cl_2_, reflux, 4 h (97% yield, 1:1.8 *E:Z* ratio); b) H_2_ (1 atm), Pd/C (30 mol%), Et_3_N (15 equiv), MeOH/CH_2_Cl_2_ (1:1), rt, 30 min (80%); c) PPTS (10 mol%), MeOH/THF (3:2), rt, 17 h (97% yield).

In conclusion, we have developed the shortest total syntheses of the macrocyclic natural products isoriccardin C and isoriccardin D (only 9 linear steps). Central to this achievement is an atom-economical intramolecular Pd­(II)-catalyzed olefination directed by the phenolic hydroxyl group. Leveraging this native directing group enables the direct assembly of the core structure without the need to use artificial auxiliary groups.

The overall yields of the macrocyclization are moderate, likely due to the inherent sensitivity of the substrates; however, they can be improved by employing more electron-deficient arylalkene acceptors, which also enable access to structurally interesting analogues. Notably, the entire synthetic route can be completed in just over 1 week, underscoring both its practicality and overall efficiency.

## Supplementary Material



## Data Availability

The data underlying this study are available in the published article and its Supporting Information.

## Notes

Experimental details and characterization data for all new compounds (PDF)
