# Supplementary material for: Total Synthesis of Isoriccardin C and Isoriccardin D Based on a Hydroxyl-Directed Palladium-Catalyzed Intramolecular C–H Alkenylation
Source: Org Lett. 2026 Apr 6;28(15):4836–40. doi: 10.1021/acs.orglett.6c00911 (PMC13097244; doi:10.1021/acs.orglett.6c00911)

Supporting Information

Total Synthesis of Isoriccardin C and Isoriccardin D based on a Hydroxyl-directed Palladium-catalyzed intramolecular C-H alkenylation

Pablo Losada, José Luis Mascareñas*, Moisés Gulías*

e-mail: joseluis.mascarenas@usc.es, moises.gulias@usc.es

Centro Singular de Investigación en Química Biológica y Materiales Moleculares (CiQUS) and Departamento de Química Orgánica, Universidad de Santiago de Compostela, 15782 Santiago de Compostela, Spain.

Table of contexts

1. General experimental information 1

2. Synthesis of Isoriccardin C 2

4-iodo-3-((tetrahydropyran-2-yl)oxy)benzaldehyde (1).......... 2

2'-methoxy-2-((tetrahydropyran-2-yl)oxy)-[1,1'-biphenyl]-4-carbaldehyde (2).......... 3

2-((4-(4-bromophenethyl)-2'-methoxy-[1,1'-biphenyl]-2-yl)oxy)tetrahydropyran (4) 4

2-methoxy-5-vinylphenol (S2).......... 5

2-((2'-methoxy-4-(4-(2-methoxy-5-vinylphenoxy)phenethyl)-[1,1'-biphenyl]-2-yl)oxy)tetrahydropyran (5) 6

2'-methoxy-4-(4-(2-methoxy-5-vinylphenoxy)phenethyl)-[1,1'-biphenyl]-2-ol (6).......... 7

(E)-7,8-dehydroisoriccardin C 3,12-dimethyl ether (7) 8

Isoriccardin C (8).......... 11

3. Synthesis of Isoriccardin D 12

4'-methoxy-2-((tetrahydropyran-2-yl)oxy)-[1,1'-biphenyl]-4-carbaldehyde (9).......... 12

2-((4-(4-bromophenethyl)-4'-methoxy-[1,1'-biphenyl]-2-yl)oxy)tetrahydropyran (10) 13

2-((4'-methoxy-4-(4-(2-methoxy-5-vinylphenoxy)phenethyl)-[1,1'-biphenyl]-2-yl) oxy)tetrahydropyran (11) 14

4'-methoxy-4-(4-(2-methoxy-5-vinylphenoxy)phenethyl)-[1,1'-biphenyl]-2-ol (12).......... 15

(E)-7,8-dehydroisoriccardin D 3,12-dimethyl ether (13) 16

Isoriccardin D (14).......... 18

4. Synthesis of nitro derivate of Isoriccardin C 19

(4-benziloxybenzyl)triphenylphosphomium bromide (S3) 19

4-(2-(2'-methoxy-2-((tetrahydropyran-2-yl)oxy)-[1,1'-biphenyl]-4-yl)ethyl)phenol.......... 20

3-fluoro-4-nitrostyrene (S4) 21

2-((2'-methoxy-4-(4-(2-nitro-5-vinylphenoxy)phenethyl)-[1,1'-biphenyl]-2-yl)oxy) tetrahydropyran (16).......... 22

2'-methoxy-4-(4-(2-nitro-5-vinylphenoxy)phenethyl)-[1,1'-biphenyl]-2-ol (17) 23

(E)-12-nitro-7,8-dehydroisoriccardin C 3-methyl ether (18).......... 24

5. NMR Spectra.......... 26

1. General experimental information

Dry solvents were obtained from Acros Organics, Extra Dry over Molecular Sieves, and used without further purification. $Pd(OAc)_2$ (98%) [3375-31-1] was obtained from Strem. All other chemicals were purchased from Sigma Aldrich, BLDpharm, Fluorochem, TCI chemicals, Alfa Aesar, abcr GmbH, Acros Organics and Fluka; and were used as received. Inert-atmosphere reactions were carried out with dry solvents in flame-dried flasks. The abbreviation “rt” refers to a temperature between 20-25 ºC. Reaction mixtures were stirred using Teflon-coated magnetic stir bars. Thin layer chromatography (TLC) was carried out on pre-coated silica gel F_{254} plates with visualization under UV light or by dipping the plate into *p*-anisaldehyde solution followed by heating. Column chromatography was performed on silica gel (40-60 µm). NMR data was collected on Varian Mercury 300 MHz or Bruker AVIII 500 MHz spectrometers. Chemical shifts are given in ppm (δ) and are referenced to the residual solvent signal ($CHCl_3$, CH_2Cl_2 or DMSO). NMR data was analyzed using MestreNova NMR data processing software (http://mestrelab.com/). High Resolution Mass Spectra (HRMS) were performed at the CACTUS facility of the University of Santiago de Compostela on a Bruker micrOTOF spectrometer. Enantiomeric ratios (er) were determined on an Agilent HPLC 1100 Series using commercially available chiral columns. X-ray crystallographic analysis of compounds **7**, **13** and **18** was performed at the CACTUS facility of the University of Santiago de Compostela on a Bruker D8 Venture or Rigaku synergy S diffractometer with Cu radiation at 100 K.

2. Synthesis of Isoriccardin C

4-iodo-3-((tetrahydropyran-2-yl)oxy)benzaldehyde (1)

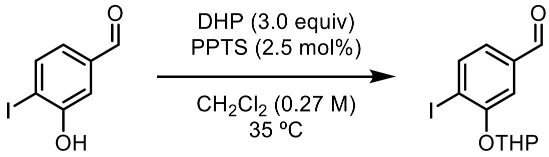

1

To a suspension of 3-hydroxy-4-iodobenzaldehyde (2.00 g, 8.06 mmol, 1.00 equiv) and PPTS (51 mg, 2.5 mol%) in CH_2Cl_2 (30 mL), DHP (2.2 mL, 24.2 mmol, 3.00 equiv) was dropwise added and the mixture was stirred at 35 ºC (aluminum heating block) under argon for 22 h. The reaction was quenched with sat. aq. $NaHCO_3$ and the layers were separated. The aqueous phase was extracted with CH_2Cl_2 (x2) and the combined organic phase was dried over Na_2SO_4 and concentrated under reduced pressure. The resulting residue was purified by flash column chromatography (AcOEt/hexane 5:95 to 6:94) to afford 2.34 g (87%) of **1** as a light-yellow oil that spontaneously crystallizes upon standing. **R_f**: 0.50 (AcOEt/hexane 15:85, brown in p-anisaldehyde). **^{1}H NMR** (500 MHz, $CDCl_3$) δ: 9.92 (s, 1H), 7.97 (d, *J* = 7.8 Hz, 1H), 7.52 (d, *J* = 1.7 Hz, 1H), 7.22 (dd, *J* = 8.0, 1.8 Hz, 1H), 5.64 (t, *J* = 3.0 Hz, 1H), 3.83 (td, *J* = 11.2, 2.9 Hz, 1H), 3.66 – 3.61 (m, 1H), 2.20 – 2.10 (m, 1H), 2.03 – 1.98 (m, 1H), 1.90 (dddd, *J* = 13.6, 12.0, 4.5, 3.0 Hz, 1H), 1.77 – 1.69 (m, 2H), 1.67 – 1.61 (m, 1H). **^{13}C NMR** (126 MHz, $CDCl_3$) δ: 191.5 (CHO), 156.4 (C), 140.2 (CH), 137.9 (C), 124.3 (CH), 114.4 (CH), 96.9 (CHO_2), 96.3 (C-I), 62.0 (CH_2O), 30.2 (CH_2), 25.2 (CH_2), 18.4 (CH_2). **HRMS**: Molecular peak could not be found for this compound.

2'-methoxy-2-((tetrahydropyran-2-yl)oxy)-[1,1'-biphenyl]-4-carbaldehyde (2)

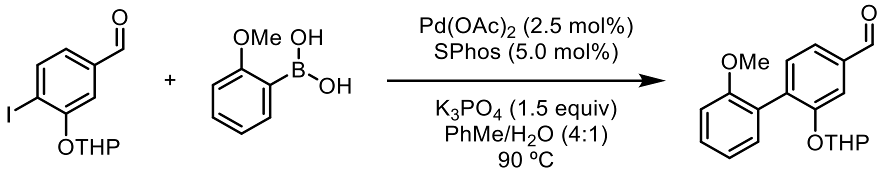

1 (1.2 equiv) **2**

A solution of **1** (3.88 g, 11.7 mmol, 1.00 equiv), 2-methoxyphenylboronic acid (2.13 g, 14.0 mmol, 1.20 equiv) and K_3PO_4 (3.72 g, 17.5 mmol, 1.50 equiv) in PhMe (23.4 mL), and water (5.8 mL) was degassed by bubbling an argon steam for 5 min. $Pd(OAc)_2$ (66 mg, 2.5 mol%) and SPhos (240 mg, 5.0 mol%) were added and the mixture was stirred at 90 ºC (aluminum heating block) under argon for 2.5 h. After cooling to rt, the reaction mixture was partitioned between AcOEt and water. The layers were separated, and the aqueous phase was extracted with AcOEt (x2). The combined organic phase was washed with brine, dried over Na_2SO_4, and concentrated under reduced pressure. The resulting residue was purified by flash column chromatography (AcOEt/hexane 10:90 to 12:88) to afford 3.37 g (92%) of **2** as a brown foamy wax. **R_f**: 0.30 (AcOEt/hexane 10:90, dark brown in p-anisaldehyde). **^{1}H NMR** (300 MHz, $CDCl_3$) δ: 10.00 (s, 1H), 7.72 (s, 1H), 7.56 (d, *J* = 7.7 Hz, 1H), 7.43 (d, *J* = 7.6 Hz, 1H), 7.36 (t, *J* = 7.8 Hz, 1H), 7.25 (d, *J* = 7.5 Hz, 1H), 7.06 – 6.95 (m, 2H), 5.48 (s, 1H), 3.86 – 3.71 (m, 4H), 3.59 (d, *J* = 11.1 Hz, 1H), 1.78 – 1.43 (m, 6H). **^{13}C NMR** (75 MHz, $CDCl_3$) δ: 192.1 (CHO), 156.9 (C), 155.2 (C), 137.0 (C), 135.7 (C), 132.1 (CH), 131.2 (CH), 129.3 (CH), 126.9 (C), 123.3 (CH), 120.3 (CH), 115.4 (CH), 110.6 (CH), 96.5 (CHO_2), 61.7 (CH_2O), 55.5 (OCH_3), 30.2 (CH_2), 25.3 (CH_2), 18.3 (CH_2). **HRMS** (APCI+) *m/z* calcd. for $C_{14}H_{13}O_3$ [M-THP+2H]: 229.0859; found: 229.0855.

2-((4-(4-bromophenethyl)-2'-methoxy-[1,1'-biphenyl]-2-yl)oxy)tetrahydropyran (4)

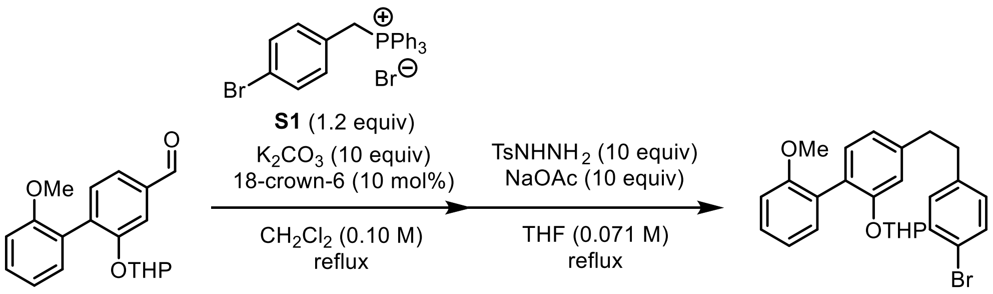

2 **4**

A mixture of **2** (1.06 g, 3.39 mmol, 1.00 equiv), **S1** (2.09 g, 4.07 mmol, 1.20 equiv), K_2CO_3 (4.69 g, 33.9 mmol, 10.0 equiv) and 18-crown-6 (90 mg, 10 mol%) in CH_2Cl_2 (34 mL) was refluxed (aluminum heating block) under argon atmosphere for 0.5 h. After cooling to rt, the reaction mixture was partitioned between water and CH_2Cl_2. The layers were separated, and the aqueous phase was extracted with CH_2Cl_2 (x2). The combined organic phase was dried over Na_2SO_4 and concentrated under reduced pressure. The resulting residue was purified by flash column chromatography (AcOEt/hexane 5:95) to afford 1.38 g (87%, E/Z ~1:1.6) of **3** as a cream foam.

A suspension of **3** (620 mg, 1.33 mmol, 1.00 equiv), $TsNHNH_2$ (2.48 g, 13.3 mmol, 10.0 equiv) and NaOAc (1.09 g, 13.3 mmol, 10.0 equiv) in THF (19.0 mL) was refluxed (aluminum heating block at 70 ºC) under air atmosphere for 24 h. As NMR showed partial conversion, solid Na_2CO_3 was added to destroy the formed AcOH (that can cleave the THP moiety) and the mixture was refluxed for further 3 days. After cooling to rt, the THF was removed under reduced pressure, and the resulting residue was diluted with water and extracted with Et_2O (x4). The combined organic phase was dried over Na_2SO_4 and concentrated under reduced pressure. The resulting residue was dissolved in the minimum amount of CH_2Cl_2 and passed through a silica-gel pad eluting with CH_2Cl_2 to remove excess $TsNHNH_2$. The filtrate was concentrated, and the resulting residue was purified by flash column chromatography (AcOEt/hexane 5:95 to 8:92) to afford 484 mg (78%) of **4** as a foamy oil that spontaneously crystallized upon standing. **R_f**: 0.50 (AcOEt/hexane 10:90, brown to cream in p-anisaldehyde). **^{1}H NMR** (500 MHz, $CDCl_3$) δ: 7.43 – 7.37 (m, 2H), 7.31 (ddd, J = 8.3, 7.3, 1.8 Hz, 1H), 7.24 (dd, overlaps with $CHCl_3$, 1H), 7.18 (d, J = 7.6 Hz, 1H), 7.14 – 7.05 (m, 2H), 7.02 (d, J = 1.8 Hz, 1H), 6.99 (td, J = 7.4, 1.2 Hz, 1H), 6.95 (dd, J = 8.3, 1.2 Hz, 1H), 6.87 (dd, J = 7.7, 1.8 Hz, 1H), 5.30 (t, J = 2.7 Hz, 1H), 3.83 (td, J = 11.0, 2.8 Hz, 1H), 3.77 (s, 3H), 3.57 – 3.51 (m, 1H), 2.92 (s, 4H), 1.68 – 1.56 (m, 4H), 1.52 – 1.43 (m, 2H). **^{13}C NMR** (126 MHz, $CDCl_3$) δ: 157.2 (C), 154.6 (C), 142.0 (C), 141.0 (C), 131.8 (CH), 131.5 (2xCH), 131.3 (CH), 130.4 (2xCH),

128.5 (CH), 128.1 (C), 126.8 (C), 121.6 (CH), 120.2 (CH), 119.8 (C), 115.9 (CH), 110.6 (CH), 96.8 (CHO_2), 61.6 (OCH_2), 55.6 (OCH_3), 37.9 (Ar-CH_2), 37.2 (Ar-CH_2), 30.5 (CH_2), 25.5 (CH_2), 18.4 (CH_2). **HRMS** (APCI+) *m/z* calcd. for $C_{21}H_{20}{}^{79}BrO_2$ [M-THP+2H]: 383.0641; found: 383.0649.

2-methoxy-5-vinylphenol (S2)

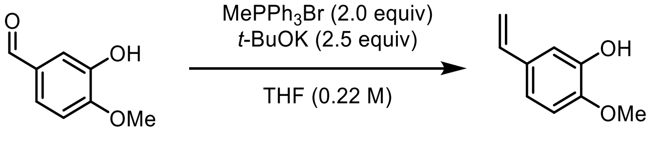

S2

$MePPh_3Br$ (9.39 g, 26.3 mmol, 2.00 equiv) and *t*-BuOK (3.67 g, 32.9 mmol, 2.50 equiv) were dried under high vacuum in a 250 mL RBF for 5 min. Then, argon atmosphere was introduced and THF (60 mL) was injected. The resulting orange suspension was stirred at rt for 1 h. Isovanillin (2.00 g, 13.1 mmol, 1.00 equiv) was added at once and the mixture was stirred at rt for 2 h. The reaction was quenched with sat. aq. NH_4Cl, followed by water. Most part of the THF was removed under reduced pressure and the resulting aqueous residue was extracted with CH_2Cl_2 (x3). The combined organic phase was dried over $MgSO_4$ and concentrated under reduced pressure. The resulting residue was purified by flash column chromatography (AcOEt/hexane 5:95 to 10:90) to afford 1.49 g (76%) of **S2** as a white solid. **R_f**: 0.30 (AcOEt/hexane 10:90, violet in p-anisaldehyde). **^{1}H NMR** (300 MHz, $CDCl_3$) δ: 7.05 (d, *J* = 2.1 Hz, 1H), 6.88 (dd, *J* = 8.3, 2.0 Hz, 1H), 6.80 (d, *J* = 8.2 Hz, 1H), 6.62 (dd, *J* = 17.5, 10.9 Hz, 1H), 5.60 (d, *J* = 16.5 Hz, 1H), 5.13 (d, *J* = 10.8 Hz, 1H), 3.89 (s, 3H). The spectroscopic data matches with the reported in the literature.[1]

[1] Wuensch, C.; Pavkov-Keller, T.; Steinkellner, G.; Gross, J.; Fuchs, M.; Hromic, A.; Lyskowski, A.; Fauland, K.; Gruber, K.; Glueck, S. M.; Faber, K. Regioselective Enzymatic β-Carboxylation of para-Hydroxy- styrene Derivatives Catalyzed by Phenolic Acid Decarboxylases. *Adv. Synth. Catal.* **2015**, *357*, 1909–1918.

2-((2'-methoxy-4-(4-(2-methoxy-5-vinylphenoxy)phenethyl)-[1,1'-biphenyl]-2-yl)oxy)tetrahydropyran (5)

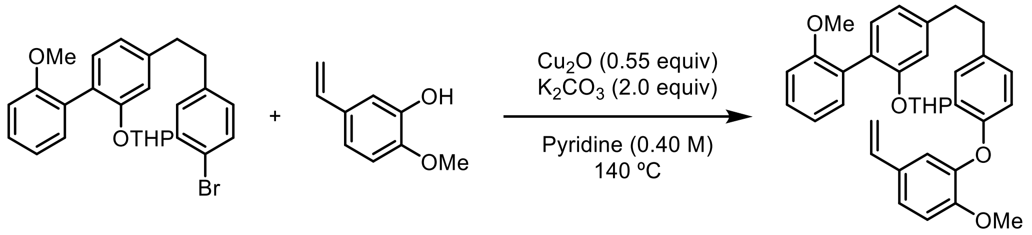

4 **S2** (1.2 equiv) **5**

S2 (18 mg, 0.12 mmol, 1.2 equiv), Cu_2O (8.0 mg, 0.055 mmol, 0.55 equiv) and K_2CO_3 (28 mg, 0.20 mmol, 2.0 equiv) were weighed and added to an oven-dried sealed tube. After three vacuum-argon cycles, a solution of **4** (47 mg, 0.10 mmol, 1.0 equiv) in dry pyridine (0.25 mL) was injected. The tube was sealed and stirred at 140 ºC in an oil bath for 22.5 h. After cooling to rt, the mixture was diluted with AcOEt and filtered through a Florisil pad, washing the tube and the pad with AcOEt for several times. The filtrate was concentrated under reduced pressure, and the resulting residue was purified by flash column chromatography (AcOEt/hexane 10:90) to afford 36 mg (66%) of **5** as a white foam. **R_f**: 0.25 (AcOEt/hexane 10:90, violet in p-anisaldehyde). **^{1}H NMR** (500 MHz, $CDCl_3$) δ: 7.31 (ddd, *J* = 8.2, 7.4, 1.8 Hz, 1H), 7.26 (dd, *J* = 7.5, 1.7 Hz, 1H), 7.18 (d, *J* = 7.7 Hz, 1H), 7.18 (d, *J* = 7.7 Hz, 1H), 7.17 – 7.11 (m, 3H), 7.09 – 7.04 (m, 2H), 6.99 (td, *J* = 7.4, 1.1 Hz, 1H), 6.97 – 6.93 (m, 2H), 6.93 – 6.87 (m, 3H), 6.59 (dd, *J* = 17.6, 10.9 Hz, 1H), 5.55 (dd, *J* = 17.6, 1.0 Hz, 1H), 5.34 (t, *J* = 2.7 Hz, 1H), 5.12 (dd, *J* = 10.8, 0.9 Hz, 1H), 3.86 (s, 3H), 3.85 – 3.80 (m, 1H), 3.76 (s, 3H), 3.57 – 3.52 (m, 1H), 2.93 (s, 4H), 1.70 – 1.56 (m, 4H), 1.50 – 1.43 (m, 2H). **^{13}C NMR** (126 MHz, $CDCl_3$) δ: 157.2 (C), 156.0 (C), 154.6 (C), 151.3 (C), 145.6 (C), 142.6 (C), 136.4 (C), 136.0 (CH), 131.8 (CH), 131.3 (C), 131.2 (CH), 129.6 (2xCH), 128.4 (CH), 128.2 (C), 126.7 (C), 122.8 (CH), 121.6 (CH), 120.2 (CH), 118.3 (CH), 117.4 (2xCH), 115.9 (CH), 112.8 (CH), 112.6 (CH_2), 110.6 (CH), 96.7 (CHO_2), 61.6 (OCH_2), 56.3 (OCH_3), 55.6 (OCH_3), 38.2 (Ar-CH_2), 37.1 (Ar-CH_2), 30.5 (CH_2), 25.5 (CH_2), 18.4 (CH_2). **HRMS** (APCI+) *m/z* calcd. for $C_{35}H_{37}O_5$ [M+H]: 537.2636; found: 537.2637.

2'-methoxy-4-(4-(2-methoxy-5-vinylphenoxy)phenethyl)-[1,1'-biphenyl]-2-ol (6)

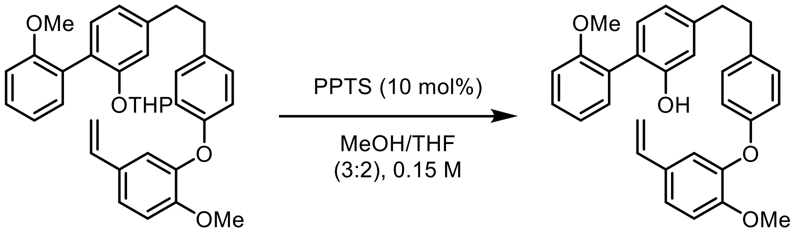

5 **6**

A solution of **5** (235 mg, 438 µmol, 1.0 equiv) and PPTS (30 mg, 10 mol%) in MeOH (1.8 mL) and THF (1.1 mL) was stirred at rt under air for 16 h. The reaction mixture was concentrated under reduced pressure, and the resulting residue was purified by flash column chromatography (AcOEt/hexane 12:88) to afford 180 mg (91%) of **6** as a white foam. **R_f**: 0.50 (AcOEt/hexane 30:70, purplish blue in p-anisaldehyde). **^{1}H NMR** (300 MHz, $CDCl_3$) δ: 7.42 – 7.32 (m, 2H), 7.19 – 7.03 (m, 7H), 6.97 – 6.83 (m, 5H), 6.60 (dd, *J* = 17.4, 10.9 Hz, 1H), 6.25 (s, 1H), 5.55 (dt, *J* = 17.5, 1.0 Hz, 1H), 5.12 (dt, *J* = 10.8, 1.0 Hz, 1H), 3.91 (s, 3H), 3.86 (s, 3H), 2.96 – 2.89 (m, 4H). **^{13}C NMR** (75 MHz, $CDCl_3$) δ: 156.1 (C-O), 155.7 (C-O), 153.8 (C-O), 151.3 (C-O), 145.7 (C-O), 143.5 (C), 136.3 (C), 136.0 (CH), 132.6 (CH), 131.4 (C), 131.3 (CH), 129.6 (2xCH), 129.3 (CH), 127.3 (C), 124.0 (C), 122.8 (CH), 122.4 (CH), 121.4 (CH), 118.3 (CH), 117.5 (3xCH), 112.8 (CH), 112.6 (CH_2), 111.8 (CH), 56.4 (OCH_3), 56.3 (OCH_3), 37.8 (CH_2), 37.0 (CH_2). **HRMS** (APCI+) *m/z* calcd. for $C_{30}H_{29}O_4$ [M+H]: 453.2060; found: 453.2065.

(E)-7,8-dehydroisoriccardin C 3,12-dimethyl ether (7)

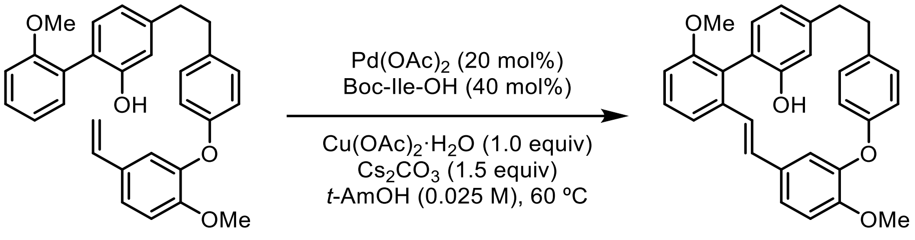

6 **7**

A mixture of **6** (45.3 mg, 0.10 mmol, 1.0 equiv), $Pd(OAc)_2$ (4.5 mg, 20 mol%), Boc-Ile-OH (9.3 mg, 40 mol%), $Cu(OAc)_2 \cdot H_2O$ (20 mg, 1.0 equiv) and Cs_2CO_3 (49 mg, 1.5 equiv) in *t*-AmOH (4.0 mL) was stirred at 60 ºC (oil bath) under argon atmosphere in a sealed tube for 6 h. After cooling to rt, the reaction mixture was filtered through a Florisil pad eluting with AcOEt. The filtrate was concentrated under reduced pressure, and the crude residue was purified by flash column chromatography (AcOEt/hexane 10:90 to 20:80) to afford 13.7 mg (30%, 53:47 er) of **7** as a white solid. $\mathbf{R_f}$: 0.35 (AcOEt/hexane 30:70, light red in p-anisaldehyde). **^{1}H NMR** (500 MHz, $CDCl_3$) δ: 7.42 (dd, *J* = 8.0, 1.1 Hz, 1H), 7.34 (t, *J* = 8.0 Hz, 1H), 7.25 (dd, overlaps with $CHCl_3$, 1H), 7.05 (dd, *J* = 8.2, 2.3 Hz, 1H), 6.99 (dd, *J* = 8.3, 2.6 Hz, 1H), 6.91 – 6.83 (m, 4H), 6.83 – 6.78 (m, 2H), 6.76 (d, *J* = 7.7 Hz, 1H), 6.55 (dd, *J* = 7.7, 1.8 Hz, 1H), 6.23 (d, *J* = 2.1 Hz, 1H), 6.18 (d, *J* = 16.2 Hz, 1H), 5.00 (s, 1H), 3.95 (s, 3H), 3.78 (s, 3H), 3.26 – 3.14 (m, 2H), 3.10 – 3.00 (m, 2H). **^{13}C NMR** (126 MHz, $CDCl_3$) δ: 157.3 (C), 153.3 (C), 152.7 (C), 150.5 (C), 148.8 (C), 141.9 (C), 138.33 (C), 137.4 (C), 131.8 (CH), 131.1 (CH), 130.4 (CH), 130.3 (C), 129.2 (CH), 127.9 (CH), 125.2 (CH), 124.2 (C), 122.4 (CH), 122.4 (CH), 122.2 (CH), 122.0 (CH), 120.3 (C), 117.6 (CH), 116.6 (CH), 111.5 (CH), 109.8 (CH), 109.7 (CH), 56.2 (OCH_3), 56.1 (OCH_3), 35.7 (CH_2), 35.1 (CH_2). **HRMS** (APCI+) *m/z* calcd. for $C_{30}H_{27}O_4$ [M+H]: 451.1904; found: 451.1897.

Enantioselectivity was determined by chiral HPLC analysis (Chiralpak IA3, IPA/hexane 15:85, 0.5 mL/min, λ=254 nm).

Racemic sample

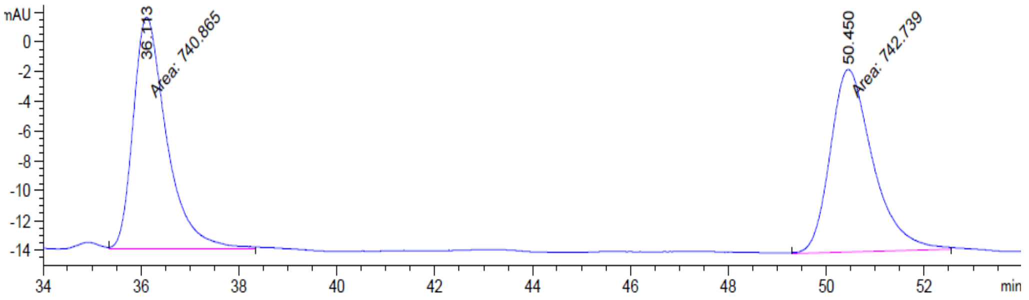

Peak #	RetTime [min]	Type	Width [min]	Area [mAU*s]	Height [mAU]	Area %
1	36.113	MM	0.7922	740.86499	15.58683	49.9368
2	50.450	MM	1.0085	742.73926	12.27464	50.0632

Enantioenriched sample

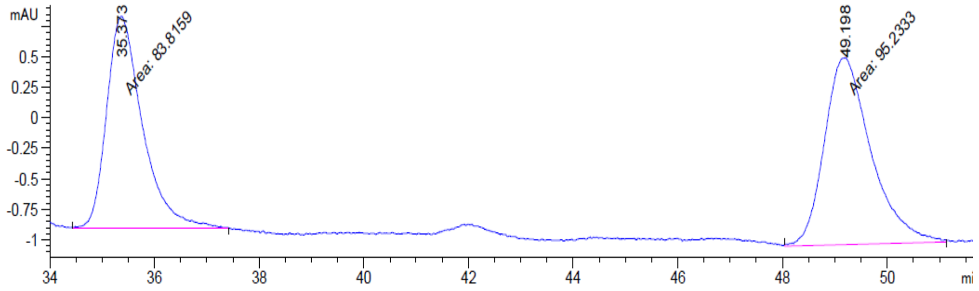

Peak #	RetTime [min]	Type	Width [min]	Area [mAU*s]	Height [mAU]	Area %
1	35.373	MM	0.7996	83.81586	1.74702	46.8116
2	49.198	MM	1.0322	95.23331	1.53774	53.1884

Crystallization of compound **7** from CH_2Cl_2/heptane afforded suitable crystals for X-ray diffraction analysis. The structure was deposited in the Cambridge Structural Database under the following deposition number: 2526910.

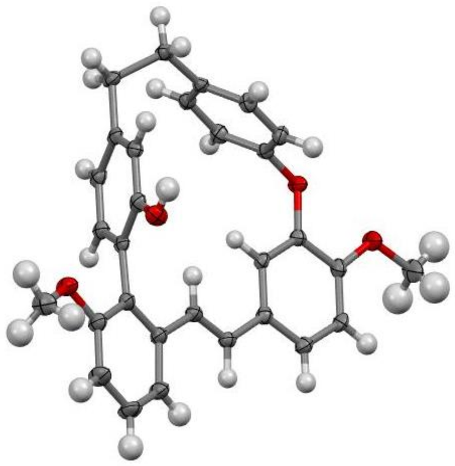

Figure S1. Thermal ellipsoid plot of compound **7** with ellipsoid contour 50% probability levels.

Table S1. Crystal parameters for compound **7**.

M_r	450.51
Crystal system, space group	Triclinic, *P*-1
Temperature (K)	100
a, *b*, *c* (Å)	9.3440 (2), 10.7513 (2), 12.7352 (3)
α, *β*, *γ* (°)	66.139 (1), 82.888 (1), 78.644 (1)
V (Å^3)	1145.73 (4)
Z	2
Radiation type	Cu *Kα*
μ (mm^{-1})	0.69
Crystal size (mm)	0.14 × 0.12 × 0.09

Isoriccardin C (8)

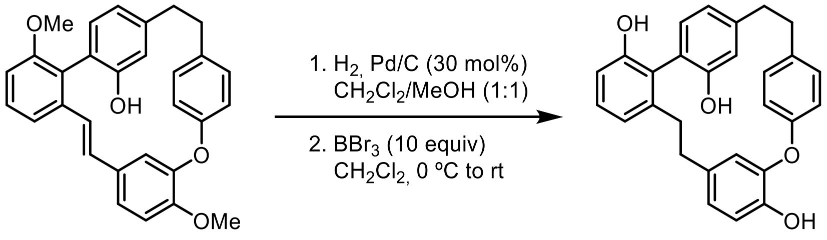

7 **8**

5% Pd/C (13.2 mg, 30 mol%) was added to a solution of **7** (16.4 mg, 0.036 mmol, 1.00 equiv) in CH_2Cl_2 (0.55 mL) and MeOH (0.55 mL) under nitrogen atmosphere. The resulting suspension was purged with hydrogen for 1 minute and then stirred under hydrogen atmosphere (balloon) for 1.5 h. The catalyst was removed by filtration through a Celite pad eluting with CH_2Cl_2. The filtrate was concentrated under reduced pressure, and the resulting solid was dissolved in CH_2Cl_2 (0.80 mL). The solution was poured under argon atmosphere and cooled to 0 ºC. BBr_3 (1.0 M in CH_2Cl_2, 0.36 mL, 0.36 mmol, 10 equiv) was dropwise added and the mixture was stirred at rt for 30 min. The reaction was quenched with water and the layers were separated. The aqueous phase was extracted with CH_2Cl_2 (x2) and AcOEt. The combined organic phase was dried over Na_2SO_4 and concentrated under reduced pressure. The resulting residue was purified by flash column chromatography (AcOEt/hexane 30:70) to afford 8.6 mg (56%, 2 steps) of **8** as a white solid. $\mathbf{R_f}$: 0.40 (AcOEt/hexane 35:65, deep red in p-anisaldehyde). **^{1}H NMR** (500 MHz, $CDCl_3$) δ: 7.28 (t, overlaps with $CHCl_3$, 1H), 7.14 (dd, *J* = 8.3, 2.2 Hz, 1H), 7.08 (dd, *J* = 8.2, 2.3 Hz, 1H), 6.96 (dd, *J* = 7.7, 1.2 Hz, 1H), 6.91 (d, *J* = 7.6 Hz, 1H), 6.89 – 6.82 (m, 4H), 6.77 (d, *J* = 1.8 Hz, 1H), 6.69 (dd, *J* = 8.1, 2.1 Hz, 1H), 6.65 (dd, *J* = 7.6, 1.7 Hz, 1H), 5.60 (d, *J* = 2.1 Hz, 1H), 5.53 (s, 1H), 4.73 (s, 1H), 4.67 (s, 1H), 3.23 – 3.15 (m, 1H), 3.15 – 3.03 (m, 3H), 2.56 – 2.46 (m, 2H), 2.37 – 2.29 (m, 1H), 2.26 – 2.19 (m, 1H). **^{13}C NMR** (126 MHz, $CDCl_3$) δ: 154.2 (C), 153.6 (C), 153.4 (C), 148.0 (C), 143.8 (C), 143.5 (C), 143.1 (C), 137.3 (C), 133.8 (C), 131.0 (CH), 130.7 (CH), 130.5 (CH), 130.3 (CH), 122.8 (CH), 121.9 (CH), 121.8 (CH), 121.8 (CH), 121.8 (CH), 120.6 (C), 117.2 (C), 116.8 (CH), 114.8 (CH), 114.7 (CH), 113.4 (CH), 38.1 (CH_2), 36.7 (CH_2), 36.2 (CH_2), 34.6 (CH_2). **HRMS** (APCI+) *m/z* calcd. for $C_{28}H_{25}O_4$ [M+H]: 425.1747; found: 425.1747.

3. **Synthesis of Isoriccardin D**

4'-methoxy-2-((tetrahydropyran-2-yl)oxy)-[1,1'-biphenyl]-4-carbaldehyde (9)

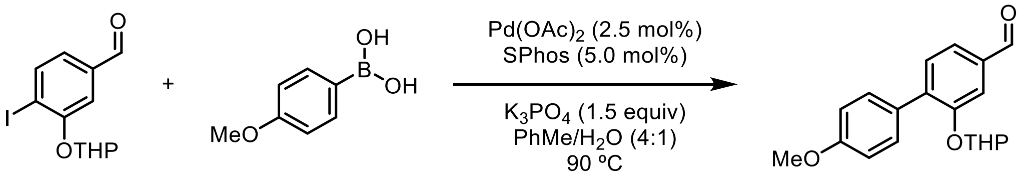

1 (1.2 equiv) **9**

A solution of **1** (6.96 g, 20.9 mmol, 1.00 equiv), 4-methoxyphenylboronic acid (3.82 g, 25.1 mmol, 1.20 equiv) and K_3PO_4 (6.67 g, 31.4 mmol, 1.50 equiv) in PhMe (41.9 mL), and water (10.5 mL) was degassed by bubbling an argon steam for 5 min. $Pd(OAc)_2$ (118 mg, 2.5 mol%) and SPhos (430 mg, 5.0 mol%) were added and the mixture was stirred at 90 ºC (aluminum heating block) under argon for 0.5 h. After cooling to rt, the reaction mixture was partitioned between AcOEt and water. The layers were separated, and the aqueous phase was extracted with AcOEt (x2). The combined organic phase was washed with brine, dried over Na_2SO_4, and concentrated under reduced pressure. The resulting residue was purified by flash column chromatography (AcOEt/hexane 8:92 to 12:88) to afford 5.56 g (85%) of **9** as a brown foamy dense oil. **R$_f$**: 0.30 (AcOEt/hexane 15:85, dark brown in p-anisaldehyde). **^{1}H NMR** (500 MHz, $CDCl_3$) δ: 9.98 (s, 1H), 7.71 (d, *J* = 1.5 Hz, 1H), 7.57 – 7.54 (m, 3H), 7.48 (d, *J* = 7.8 Hz, 1H), 6.99 – 6.96 (m, 2H), 5.52 (t, *J* = 2.8 Hz, 1H), 3.87 (s, 3H), 3.78 (td, *J* = 11.0, 3.0 Hz, 1H), 3.64 – 3.59 (m, 1H), 1.88 – 1.78 (m, 3H), 1.72 – 1.52 (m, 3H). **^{13}C NMR** (126 MHz, $CDCl_3$) δ: 191.9 (CHO), 159.5 (C), 154.4 (C), 137.7 (C), 136.4 (C), 131.2 (CH), 130.9 (2xCH), 129.9 (C), 124.0 (CH), 116.0 (CH), 113.6 (2xCH), 96.9 (CHO_2), 62.1 (CH_2O), 55.4 (OCH_3), 30.3 (CH_2), 25.3 (CH_2), 18.7 (CH_2). **HRMS** (APCI+) *m/z* calcd. for $C_{14}H_{13}O_3$ [M-THP+2H]: 229.0859; found: 229.0852.

2-((4-(4-bromophenethyl)-4'-methoxy-[1,1'-biphenyl]-2-yl)oxy)tetrahydropyran (10)

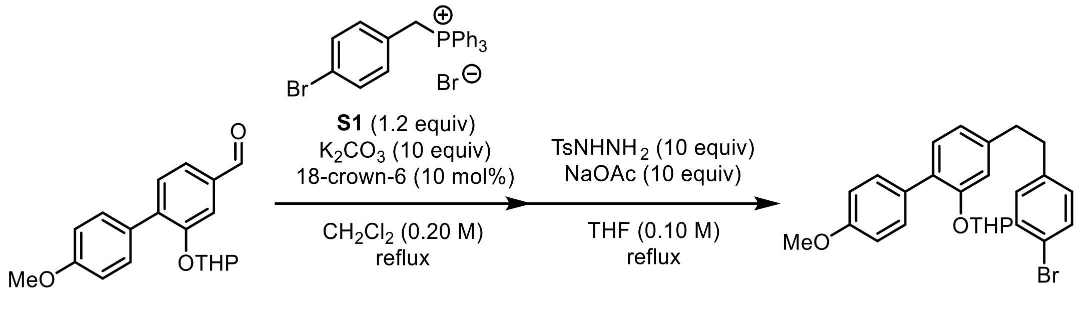

A solution of **9** (5.54 g, 17.7 mmol, 1.0 equiv), **S1** (10.7 g, 20.9 mmol, 1.2 equiv), K_2CO_3 (24.5 g, 177 mmol, 10 equiv) and 18-crown-6 (469 mg, 10 mol%) in CH_2Cl_2 (89 mL) was refluxed (aluminum heating block) under argon atmosphere for 45 min. After cooling to rt the reaction was quenched with water. The layers were separated, and the aqueous phase was extracted with CH_2Cl_2 (x2). The combined organic phase was dried over Na_2SO_4 and concentrated under reduced pressure. The resulting residue was taken in Et_2O and stirred for 5 min. The precipitated TPPO was removed by vacuum filtration and washed with Et_2O. The filtrate was concentrated and the resulting residue was purified by flash column chromatography (AcOEt/hexane 5:95 to 7:93) to afford 7.36 g (89%, E/Z ~1:9) of the stilbene as a white foam.

A mixture of the stilbene (4.80 g, 10.3 mmol, 1.00 equiv), $TsNHNH_2$ (19.2 g, 103 mmol, 10.0 equiv) and NaOAc (8.46 mmol, 103 mmol, 10.0 equiv) in THF (103 mL) was refluxed (aluminum heating block) under air for 30 h. As NMR showed partial conversion, solid Na_2CO_3 was added to destroy the formed AcOH (that can cleave the THP moiety) and the mixture was refluxed for further 15 h. After cooling to rt, the THF was removed under reduced pressure, and the resulting residue was diluted with water and extracted with Et_2O (x4). The combined organic phase was dried over Na_2SO_4 and concentrated under reduced pressure. The resulting residue was dissolved in the minimum amount of CH_2Cl_2 and passed through a silica-gel pad eluting with CH_2Cl_2 to remove excess $TsNHNH_2$. The filtrate was concentrated, and the resulting residue was purified by flash column chromatography (AcOEt/hexane 5:95 to 10:90) to afford 4.00 g (83%) of **10** as a white solid. **R_f**: 0.50 (AcOEt/hexane 10:90, brown in p-anisaldehyde). **^{1}H NMR** (500 MHz, $CDCl_3$) δ 7.52 – 7.47 (m, 2H), 7.42 – 7.37 (m, 2H), 7.23 (d, J = 7.7 Hz, 1H), 7.09 – 7.04 (m, 2H), 6.99 (d, J = 1.8 Hz, 1H), 6.96 – 6.92 (m, 2H), 6.86 (dd, J = 7.8, 1.7 Hz, 1H), 5.33 (t, J = 3.1 Hz, 1H), 3.85 (s, 3H), 3.80 (td, J = 10.9, 2.9 Hz, 1H), 3.58 – 3.54 (m, 1H), 2.90 (s, 4H), 1.88 – 1.78 (m, 1H), 1.76 – 1.72 (m, 2H), 1.69 – 1.60 (m, 1H), 1.60 – 1.50 (m, 2H). **^{13}C NMR** (126 MHz, $CDCl_3$) δ: 158.6 (C), 153.9 (C), 141.6 (C), 140.8 (C), 131.5

(2xCH), 131.2 (C), 130.8 (2xCH), 130.6 (CH), 130.5 (2xCH), 129.2 (C), 122.2 (CH), 119.8 (C), 116.2 (CH), 113.4 (2xCH), 96.9 (CHO_2), 62.0 (OCH_2), 55.4 (OCH_3), 37.7 (Ar-CH_2), 37.3 (Ar-CH_2), 30.5 (CH_2), 25.4 (CH_2), 18.7 (CH_2). **HRMS** (APCI+) *m/z* calcd. for $C_{26}H_{28}{}^{79}BrO_3$ [M+H]: 467.1216; found: 467.1216.

2-((4'-methoxy-4-(4-(2-methoxy-5-vinylphenoxy)phenethyl)-[1,1'-biphenyl]-2-yl) oxy)tetrahydropyran (11)

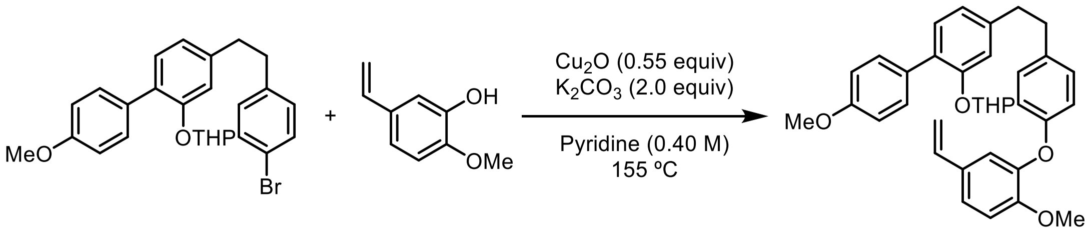

10 **S2** (1.2 equiv) **11**

10 (1.16 g, 2.48 mmol, 1.00 equiv), **S2** (447 mg, 2.97 mmol, 1.20 equiv), Cu_2O (197 g, 1.38 mmol, 0.55 equiv) and K_2CO_3 (685 mg, 4.96 mmol, 2.00 equiv) were weighed and added to an oven-dried sealed tube. After three vacuum-argon cycles, dry pyridine (6.2 mL) was injected. The tube was sealed and stirred at 155 ºC in an oil bath for 24 h. After cooling to rt, the mixture was diluted with AcOEt and filtered through a Celite pad, washing the tube and the pad with AcOEt for several times. The filtrate was concentrated under reduced pressure, and the resulting residue was purified by flash column chromatography (AcOEt/hexane 10:90 to 12:88) to afford 970 mg (73%) of **11** as a colorless foam. $\mathbf{R_f}$: 0.28 (AcOEt/hexane 15:85, violet in p-anisaldehyde). **^{1}H NMR** (500 MHz, $CDCl_3$) δ 7.52 – 7.48 (m, 2H), 7.24 (d, *J* = 7.8 Hz, 1H), 7.17 – 7.12 (m, 3H), 7.06 – 7.04 (m, 2H), 6.97 – 7.93 (m, 3H), 6.92 – 6.85 (m, 3H), 6.59 (dd, *J* = 17.5, 10.9 Hz, 1H), 5.55 (d, *J* = 17.6 Hz, 1H), 5.37 (t, *J* = 3.1 Hz, 1H), 5.12 (d, *J* = 10.9 Hz, 1H), 3.85 (s, 6H), 3.81 (td, *J* = 11.0, 2.9 Hz, 1H), 3.57 (dt, *J* = 11.3, 3.4 Hz, 1H), 2.92 (s, 4H), 1.83 (tdd, *J* = 14.7, 7.4, 4.1 Hz, 1H), 1.77 – 1.72 (m, 2H), 1.64 (tdd, *J* = 14.7, 10.2, 4.3 Hz, 1H), 1.60 – 1.50 (m, 3H). **^{13}C NMR** (126 MHz, $CDCl_3$) δ 158.6 (C), 156.0 (C), 153.9 (C), 151.3 (C), 145.6 (C), 142.2 (C), 136.2 (C), 136.0 (CH), 131.3 (C), 131.2 (C), 130.8 (2xCH), 130.5 (CH), 129.6 (2xCH), 129.1 (C), 122.8 (CH), 122.2 (CH), 118.2 (CH), 117.4 (2xCH), 116.2 (CH), 113.4 (2xCH), 112.8 (CH), 112.6 (CH_2), 96.9 (CHO_2), 61.9 (OCH_2), 56.3 (OCH_3), 55.4 (OCH_3), 38.1 (Ar-CH_2), 37.2 (Ar-CH_2), 30.5 (CH_2), 25.4 (CH_2), 18.7 (CH_2). **HRMS** (APCI+) *m/z* calcd. for $C_{35}H_{37}O_5$ [M+H]: 537.2636; found: 537.2640.

4'-methoxy-4-(4-(2-methoxy-5-vinylphenoxy)phenethyl)-[1,1'-biphenyl]-2-ol (12)

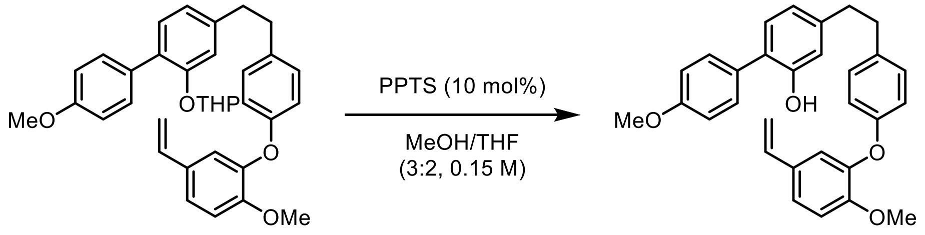

11 **12**

A solution of **11** (750 mg, 1.40 mmol, 1.0 equiv) and PPTS (95 mg, 10 mol%) in MeOH (5.6 mL) and THF (3.5 mL) was stirred at rt under air for 23 h. The reaction mixture was concentrated under reduced pressure, and the resulting residue was purified by flash column chromatography (AcOEt/hexane 15:85 to 20:80) to afford 574 mg (91%) of **12** as a colorless foamy oil that spontaneously crystallizes into a white solid. **R_f**: 0.50 (AcOEt/hexane 30:70, purplish blue in p-anisaldehyde). **R_f**: 0.25 (AcOEt/hexane 25:75, purplish blue in p-anisaldehyde). **^{1}H NMR** (500 MHz, $CDCl_3$) δ 7.40 – 7.37 (m, 2H), 7.16 – 7.12 (m, 4H), 7.05 (d, *J* = 2.1 Hz, 1H), 7.03 – 7.00 (m, 2H), 6.95 (d, *J* = 8.4 Hz, 1H), 6.92 – 6.89 (m, 2H), 6.82 – 6.79 (m, 2H), 6.60 (dd, *J* = 17.6, 10.9 Hz, 1H), 5.55 (dd, *J* = 17.5, 0.9 Hz, 1H), 5.14 (s, 1H), 5.12 (dd, *J* = 10.9, 0.8 Hz, 1H), 3.86 (s, 3H), 3.85 (s, 3H), 2.95 – 2.88 (m, 4H). **^{13}C NMR** (126 MHz, $CDCl_3$) δ 159.4 (C), 156.1 (C), 152.5 (C), 151.2 (C), 145.6 (C), 143.0 (C), 136.1 (C), 136.0 (CH), 131.3 (C), 130.4 (2xCH), 130.2 (CH), 129.6 (2xCH), 129.3 (C), 125.6 (C), 122.8 (CH), 121.1 (CH), 118.2 (CH), 117.5 (2xCH), 115.7 (CH), 114.9 (2xCH), 112.8 (CH), 112.6 (CH_2), 56.3 (OCH_3), 55.5 (OCH_3), 37.8 (CH_2), 37.0 (CH_2). **HRMS** (APCI+) *m/z* calcd. for $C_{30}H_{29}O_4$ [M+H]: 453.2060; found: 453.2061.

(E)-7,8-dehydroisoriccardin D 3,12-dimethyl ether (13)

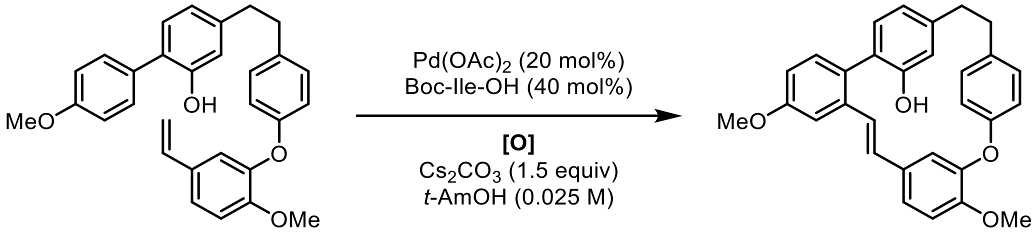

12 **13**

Table S2. Optimization for the cyclization of **12**.

Entry[a]	[O] (equiv)	Atm	T (°C)	t (h)	**13** yield (%)	**12** recov. (%)
1	$Cu(OAc)_2 \cdot H_2O$ (1.0)	air	60	3	Traces	Traces
2	-	O_2	60	24	13	39
3	$Cu(OAc)_2$ (1.5)	Ar	60	4	7	14
4	$Cu(OAc)_2$ (0.50)	O_2	60	14	5	4
**5**	**-**	**O_2**	**80**	**7**	**25**	**6**

[a] Reactions performed at 0.10 mmol scale of **12**. Yields are isolated.

Optimized procedure in entry 5

A mixture of **12** (45.3 mg, 0.10 mmol, 1.0 equiv), $Pd(OAc)_2$ (4.5 mg, 20 mol%), Boc-Ile-OH (9.3 mg, 40 mol%) and Cs_2CO_3 (49 mg, 1.5 equiv) in *t*-AmOH (4.0 mL) was stirred at 80 °C (oil bath) under oxygen atmosphere in a sealed tube for 7 h. After cooling to rt, the reaction mixture was diluted with AcOEt and filtered through a Florisil pad, washing the solids with more AcOEt. The filtrate was concentrated under reduced pressure, and the resulting residue was purified by flash column chromatography (AcOEt/hexane 12:88 to 15:85) to afford 11.2 mg (25%) of **13** as a white solid. **R_f**: 0.35 (AcOEt/hexane 30:70, light red in p-anisaldehyde). **^{1}H NMR** (500 MHz, $CDCl_3$) δ: 7.27 (d, *J* = 2.6 Hz, 1H), 7.19 (d, *J* = 8.4 Hz, 1H), 7.16 (dd, *J* = 8.3, 2.2 Hz, 1H), 7.07 (dd, *J* = 8.3, 2.2 Hz, 1H), 6.97 (dd, *J* = 8.3, 2.5 Hz, 1H), 6.93 – 6.80 (m, 6H), 6.65 (dd, *J* = 7.6, 1.7 Hz, 1H), 6.63 (d, *J* = 1.7 Hz, 1H), 6.29 (d, *J* = 2.1 Hz, 1H), 6.19 (d, *J* = 16.4 Hz, 1H), 4.62 (s, 1H), 3.95 (s, 3H), 3.88 (s, 3H), 3.17 – 3.05 (m, 4H). **^{13}C NMR** (126 MHz, $CDCl_3$) δ 159.9 (C), 152.8 (C), 152.7 (C), 150.4 (C), 149.0 (C), 141.9 (C), 137.5 (C), 137.2 (C), 131.8 (CH), 130.8 (CH), 130.7 (2xCH), 130.2 (C), 127.8 (C), 127.7 (CH), 124.8 (CH), 123.5 (C), 122.5 (CH), 122.3 (CH), 122.1 (CH), 121.3 (CH), 116.2 (CH), 114.0 (CH), 111.6 (CH), 109.9 (CH), 109.4 (CH), 56.2 (OCH_3), 55.6 (OCH_3), 35.5 (CH_2), 35.1 (CH_2). **HRMS** (APCI+) *m/z* calcd. for $C_{30}H_{27}O_4$ [M+H]: 451.1904; found: 451.1901.

Crystallization of compound **13** from CH_2Cl_2/heptane afforded suitable crystals for X-ray diffraction analysis. The structure was deposited in the Cambridge Structural Database under the following deposition number: 2526908.

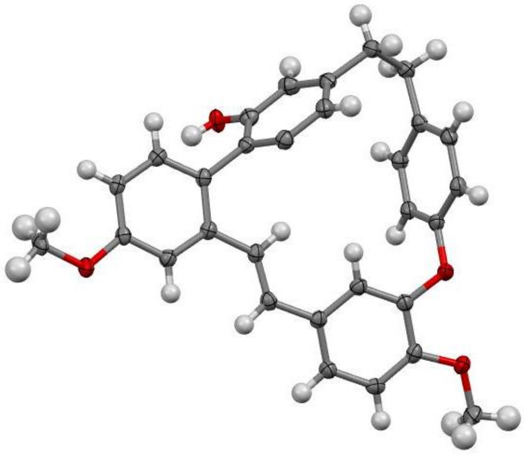

Figure S2. Thermal ellipsoid plot of compound **13** with ellipsoid contour 50% probability levels.

Table S3. Crystal parameters for compound **13**.

M_r	449.5
Crystal system, space group	Triclinic, *P-1*
Temperature (K)	100
a, *b*, *c* (Å)	10.0719 (2), 10.13563 (16), 11.2869 (4)
α, *β*, *γ* (°)	92.186 (2), 105.442 (3), 91.2044 (16)
V (Å^3)	1109.24 (6)
Z	2
Radiation type	Cu *Kα*
μ (mm^{-1})	0.71
Crystal size (mm)	0.18 × 0.12 × 0.03

Isoriccardin D (14)

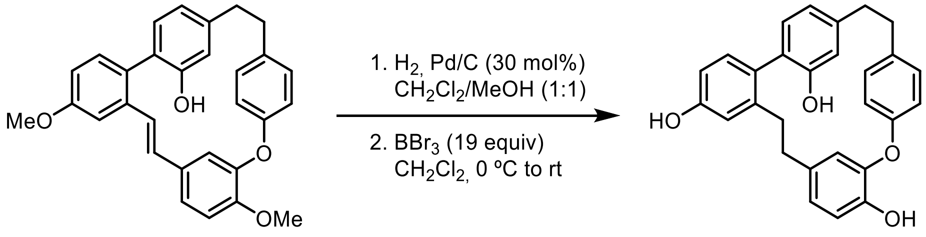

13 **14**

10% Pd/C (5.0 mg, 30 mol%) was added to a solution of **13** (7.0 mg, 0.016 mmol, 1.0 equiv) in CH_2Cl_2 (2.0 mL) and MeOH (2.0 mL) under nitrogen. The resulting suspension was purged with hydrogen and then stirred under hydrogen atmosphere (balloon) for 30 min. The catalyst was removed by filtration through a Celite pad eluting with CH_2Cl_2. The filtrate was concentrated under reduced pressure, and the resulting solid was dissolved in CH_2Cl_2 (2.0 mL). The solution was poured under argon atmosphere and cooled to 0 °C. BBr_3 (1.0 M in CH_2Cl_2, 0.30 mL, 0.30 mmol, 19 equiv) was dropwise added and the mixture was stirred at rt for 45 min. The reaction was quenched with sat. aq. $NaHCO_3$ and the mixture was partitioned between CH_2Cl_2 and water. The layers were separated, and the aqueous phase was extracted with CH_2Cl_2 (x2). The combined organic phase was dried over $MgSO_4$ and concentrated under reduced pressure. The resulting residue was purified by flash column chromatography (AcOEt/hexane 25:75) to afford 2.9 mg (44%) of **14** as a white solid. **R_f**: 0.22 (AcOEt/hexane 30:70, deep red in p-anisaldehyde). **^{1}H NMR** (500 MHz, DMSO-d_6) δ: 9.16 (s, 1H), 9.06 (s, 1H), 8.78 (s, 1H), 7.15 (dd, J = 8.5, 2.5 Hz, 1H), 7.00 (dd, J = 8.3, 2.3 Hz, 1H), 6.86 (dd, J = 8.3, 2.5 Hz, 1H), 6.78 (d, J = 8.3 Hz, 1H), 6.74 (dd, J = 8.3, 2.4 Hz, 1H), 6.71 (d, J = 3.4 Hz, 1H), 6.69 (d, J = 3.9 Hz, 1H), 6.67 (d, J = 2.6 Hz, 1H), 6.64 (dd, J = 8.1, 2.1 Hz, 1H), 6.55 (dd, J = 8.2, 2.5 Hz, 1H), 6.45 (dd, J = 7.6, 1.9 Hz, 1H), 6.41 (d, J = 1.7 Hz, 1H), 5.71 (d, J = 2.2 Hz, 1H), 3.04 – 2.96 (m, 3H), 2.91 – 2.85 (m, 1H), 2.61 – 2.54 (m, 1H), 2.36 (td, J = 12.4, 7.8 Hz, 1H), 2.21 – 2.14 (m, 2H). **^{13}C NMR** (126 MHz, DMSO-d_6) δ: 156.2 (C), 154.0 (C), 153.4 (C), 148.2 (C), 144.4 (C), 141.2 (C), 139.9 (C), 136.6 (C), 132.8 (C), 131.0 (CH), 130.6 (CH), 130.4 (CH), 130.3 (CH), 129.1 (C), 125.3 (C), 121.5 (CH), 121.1 (CH), 120.9 (CH), 119.3 (CH), 116.1 (CH), 115.8 (CH), 115.3 (CH), 115.1 (CH), 112.5 (CH), 36.9 (CH_2), 36.3 (CH_2), 35.5 (CH_2), 34.5 (CH_2). **HRMS** (APCI+) m/z calcd. for $C_{28}H_{25}O_4$ [M+H]: 425.1747; found: 425.1757.

4. Synthesis of nitro derivate of Isoriccardin C

(4-benziloxybenzyl)triphenylphosphomium bromide (S3)

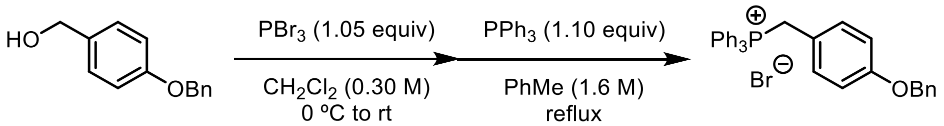

S3

To a solution of 4-benzyloxybenzyl alcohol (2.50 g, 11.7 mmol, 1.00 equiv) in CH_2Cl_2 (39 mL) at 0 ºC under argon, PBr_3 (1.2 mL, 12.3 mmol, 1.05 equiv) was dropwise added and the solution was stirred at rt for 1 h without exposing to light. The reaction was quenched with ice, followed by water. The layers were separated, and the aqueous phase was extracted with CH_2Cl_2 (x2) and Et_2O. The combined organic phase was dried over Na_2SO_4 and concentrated under reduced pressure in an opaque flask to afford the benzyl bromide as a solid that was used immediately in the following reaction.

The benzyl bromide was dissolved in PhMe (7.3 mL) and poured under argon. PPh_3 (3.37 g, 12.8 mmol, 1.10 equiv) was added and the solution was refluxed in an aluminum heating block for 2.5 h without exposing to light. After cooling to rt, the resulting slurry was diluted with Et_2O and vacuum filtered. The solid was washed with Et_2O several times and vacuum dried to afford 5.66 g (90%, 2 steps) of the title compound as a white solid. **^{1}H NMR** (300 MHz, $CDCl_3$) δ: 7.80 – 7.68 (m, 9H), 7.62 (td, *J* = 7.2, 3.6 Hz, 6H), 7.39 – 7.30 (m, 5H), 7.02 (dd, *J* = 8.9, 2.7 Hz, 2H), 6.76 – 6.70 (m, 2H), 5.34 (d, *J* = 13.9 Hz, 2H), 4.97 (s, 2H). This compound is fully characterized in the literature and the ^{1}H NMR signals match the reported values.[2]

---

[2] Bonfield, H. E.; Edge, C. M.; Reid, M.; Kennedy, A. R.; Pascoe, D. D.; Lindsay, D. M.; Valette, D. Synthesis of 2,6-trans-Tetrahydropyrans Using a Palladium-Catalyzed Oxidative Heck Redox-Relay Strategy. *Org. Lett.* **2024**, *26*, 2857.

4-(2-(2'-methoxy-2-((tetrahydropyran-2-yl)oxy)-[1,1'-biphenyl]-4-yl)ethyl)phenol

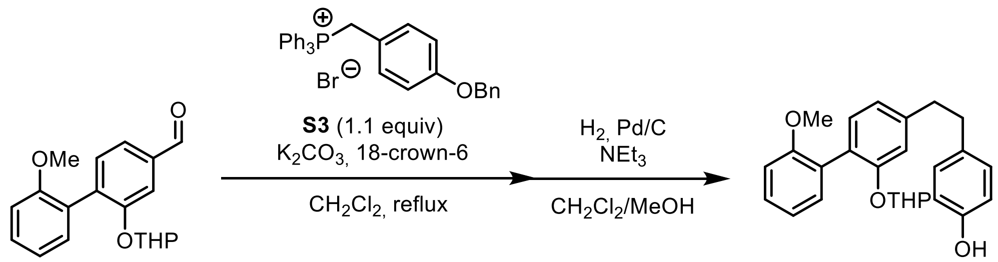

2 **15**

A suspension of **2** (3.19 g, 10.2 mmol, 1.00 equiv), **S3** (6.06 g, 11.2 mmol, 1.10 equiv), K_2CO_3 (14.1 g, 102 mmol, 10.0 equiv) and 18-crown-6 (270 mg, 10 mol%) in CH_2Cl_2 (51 mL) was refluxed (aluminum heating block) under argon atmosphere for 3.75 h. After cooling to rt, the reaction mixture was diluted with water. The layers were separated, and the aqueous phase was extracted with CH_2Cl_2 (x2). The combined organic phase was dried over Na_2SO_4 and concentrated under reduced pressure. The residue was taken in Et_2O and aged for 2 h. The precipitated TPPO was removed by vacuum filtration. The filtrate was concentrated under reduced pressure, and the resulting residue was purified by flash column chromatography (AcOEt/hexane 10:90 to 15:85) to afford 4.88 g (E/Z ~1:1.8, 97%) of the stilbene as a yellow foam.

Pd/C (10%, 3.16 g, 30 mol%) was added to a solution of the stilbene (4.88 g, 9.90 mmol, 1.00 equiv) and NEt_3 (21 mL, 149 mmol, 15 equiv) in CH_2Cl_2 (99 mL) and MeOH (99 mL) under nitrogen atmosphere. The resulting black suspension was purged with hydrogen for 1 minute and then stirred at rt under hydrogen atmosphere (balloon) for 30 min. The catalyst was removed by filtration through a Celite pad, and the solids were washed with CH_2Cl_2. The filtrate was concentrated under reduced pressure, and the resulting residue was purified by flash column chromatography (AcOEt/CH_2Cl_2 0:100 to 5:95) to afford 3.22 g (80%) of **15** as a white foam that solidifies upon storing. **R_f**: 0.60 (AcOEt/hexane 40:60, brown in p-anisaldehyde). **^{1}H NMR** (500 MHz, CD_2Cl_2) δ: 7.25 – 7.20 (m, 1H), 7.12 (dd, *J* = 7.4, 1.8 Hz, 1H), 7.04 (d, *J* = 7.6 Hz, 1H), 7.03 – 7.00 (m, 2H), 6.95 (d, *J* = 1.7 Hz, 1H), 6.91 – 6.86 (m, 2H), 6.80 (dd, *J* = 7.6, 1.7 Hz, 1H), 6.70 – 6.63 (m, 2H), 5.23 – 5.21 (m, 1H), 4.90 (s, 1H), 3.71 (td, *J* = 10.7, 2.8 Hz, 1H), 3.67 (s, 3H), 3.46 – 3.42 (m, 1H), 2.80 (s, 4H), 1.61 – 1.44 (m, 6H), 1.41 – 1.34 (m, 2H). **^{13}C NMR** (126 MHz, CD_2Cl_2) δ: 157.5 (C), 154.9 (C), 154.3 (C), 143.0 (C), 134.5 (C), 131.9 (CH), 131.5 (CH), 129.9 (2xCH), 128.8 (CH), 128.4 (C), 127.0 (C), 121.8 (CH), 120.4 (CH), 116.1 (CH), 115.5 (2xCH), 110.8 (CH), 97.1 (CHO_2), 62.1 (CH_2O), 55.7 (OCH_3), 38.5 (Ar-CH_2), 37.1 (Ar-CH_2), 30.8 (CH_2), 25.7 (CH_2), 18.9 (CH_2). **HRMS** (APCI+) *m/z* calcd. for $C_{26}H_{29}O_4$ [M+H]: 405.2060; found: 405.2066. **Notes:** Compound is unstable in chloroform due to its acidic character, observing THP cleavage and exchange.

3-fluoro-4-nitrostyrene (S4)

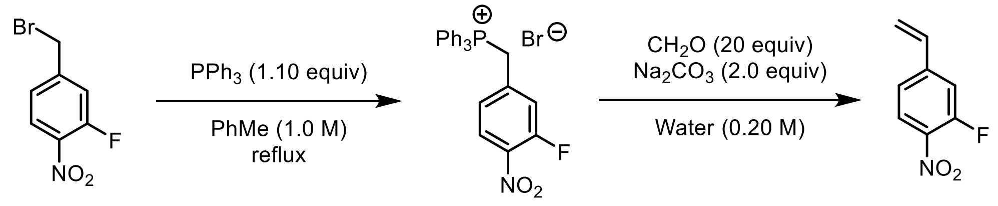

S4

To a solution of 3-fluoro-4-nitrobenzyl bromide (526 mg, 2.25 mmol, 1.00 equiv) in PhMe (2.2 mL) under argon, PPh_3 (648 mg, 2.47 mmol, 1.10 equiv) was added and the solution was refluxed (aluminum heating block) for 3 h. After cooling to rt, the resulting paste was diluted with Et_2O, crushed and vacuum filtered. The solid was washed with Et_2O several times and vacuum dried to afford 1.04 g (93%) of the phosphonium salt as a white solid. ^{1}**H NMR** (300 MHz, $CDCl_3$) δ: 7.90 – 7.69 (m, 10H), 7.68 – 7.60 (m, 6H), 7.33 (d, J = 9.0 Hz, 1H), 7.22 (d, J = 11.6 Hz, 1H), 6.05 (d, J = 15.7 Hz, 2H). ^{19}**F NMR** (471 MHz, $CDCl_3$) δ: -116.5 (m).

To a solution of Na_2CO_3 (2.08 g, 19.6 mmol, 2.00 equiv) in water (49 mL) under argon, the phosphonium bromide (4.87 g, 9.81 mmol) was added. The resulting suspension was stirred until all the salt turned red (5 min). Formaldehyde (40% in water, 15 mL, 196 mmol, 20.0 equiv) was added and the mixture was stirred until the red color faded out. CH_2Cl_2 was added, and the mixture was vigorously stirred for 25 min before the layers were separated. The aqueous phase was extracted with CH_2Cl_2, and the combined organic extract was dried over Na_2SO_4 and concentrated under reduced pressure. The resulting residue was purified by flash column chromatography (AcOEt/hexane 5:95) to afford 1.49 g (91%) of **S4** as a yellow oil. $\mathbf{R_f}$: 0.45 (AcOEt/hexane 10:90, colorless in p-anisaldehyde). ^{1}**H NMR** (500 MHz, $CDCl_3$) δ: 8.04 (t, J = 8.1 Hz, 1H), 7.30 – 7.29 (m, 1H), 7.29 – 7.27 (m, 1H), 6.72 (dd, J = 17.5, 10.9 Hz, 1H), 5.93 (d, J = 17.5 Hz, 1H), 5.55 (d, J = 10.9 Hz, 1H). ^{13}**C NMR** (126 MHz, $CDCl_3$) δ: 156.1 (d, J = 265 Hz, C-F), 145.6 (d, J = 8.6 Hz, C), 136.3 (C), 134.2 (d, J = 2.3 Hz, CH), 126.7 (d, J = 2.7 Hz, CH), 122.2 (d, J = 4.1 Hz, CH), 120.0 (CH_2), 115.64 (d, J = 21.4 Hz, CH). ^{19}**F NMR** (471 MHz, $CDCl_3$) δ: -117.1. **HRMS** (APCI+) *m/z* calcd. for $C_8H_7FNO_2$ [M+H]: 168.0455; found: 168.0457.

2-((2'-methoxy-4-(4-(2-nitro-5-vinylphenoxy)phenethyl)-[1,1'-biphenyl]-2-yl)oxy) tetrahydropyran (16)

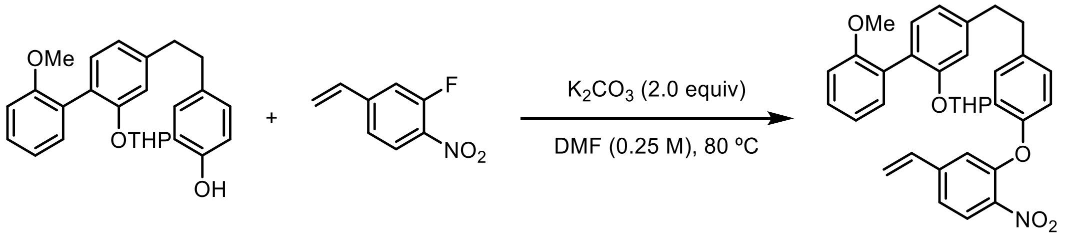

15 **S4** (1.1 equiv) **16**

A mixture of **15** (2.00 g, 4.94 mmol, 1.00 equiv), **S4** (0.909 g, 5.44 mmol, 1.10 equiv) and K_2CO_3 (1.37 g, 9.89 mmol, 2.00 equiv) in DMF (20 mL) was stirred at 80 ºC (oil bath) under argon for 2.5 h. After cooling to rt, the reaction mixture was partitioned between AcOEt and water. The layers were separated, and the aqueous phase was extracted with AcOEt (x2). The combined organic extract was dried over Na_2SO_4 and concentrated under reduced pressure. The resulting residue was purified by flash column chromatography (AcOEt/hexane 10:90 to 20:80) to afford 1.96 g (71%) of **16** as light-yellow fluffy solid. **R_f**: 0.40 (AcOEt/hexane 20:80, green in p-anisaldehyde). **^{1}H NMR** (500 MHz, CD_2Cl_2) δ: 7.95 (d, J = 8.5 Hz, 1H), 7.32 (ddd, J = 8.3, 7.4, 1.8 Hz, 1H), 7.30 – 7.27 (m, 2H), 7.25 (dd, J = 1.8, 0.6 Hz, 1H), 7.22 (dd, J = 1.8, 0.6 Hz, 1H), 7.15 (d, J = 7.6 Hz, 1H), 7.06 (d, J = 1.7 Hz, 1H), 7.02 (d, J = 1.8 Hz, 1H), 7.01 – 6.99 (m, 2H), 6.97 (dd, J = 7.6, 1.1 Hz, 1H), 6.90 (dd, J = 7.7, 1.7 Hz, 1H), 6.65 (ddd, J = 17.5, 10.9, 0.5 Hz, 1H), 5.80 (dd, J = 17.6, 0.6 Hz, 1H), 5.42 (d, J = 10.9 Hz, 1H), 5.33 – 5.31 (m, 1H), 3.81 (td, J = 10.5, 2.8 Hz, 1H), 3.76 (s, 3H), 3.55 – 3.51 (m, 1H), 3.02 – 2.94 (m, 4H), 1.68 – 1.56 (m, 4H), 1.51 – 1.44 (m, 2H). **^{13}C NMR** (126 MHz, CD_2Cl_2) δ: 157.5 (C), 155.0 (C), 154.3 (C), 151.8 (C), 144.4 (C), 142.7 (C), 140.4 (C), 138.8 (C), 135.1 (CH), 131.9 (CH), 131.6 (CH), 130.5 (2xCH), 128.8 (CH), 128.3 (C), 127.1 (C), 126.6 (CH), 121.7 (CH), 120.9 (CH), 120.4 (CH), 119.4 (2xCH), 118.9 (CH_2), 118.4 (CH), 116.1 (CH), 110.8 (CH), 97.1 (CHO_2), 62.0 (CH_2O), 55.7 (OCH_3), 38.2 (Ar-CH_2), 37.4 (Ar-CH_2), 30.8 (CH_2), 25.7 (CH_2), 18.9 (CH_2). **HRMS** (APCI+) *m/z* calcd. for $C_{29}H_{26}NO_5$ [M-THP+2H]: 468.1805; found: 468.1808. **Notes:** Compound is unstable in chloroform due to its acidic character, cleavage of the THP moiety was observed.

2'-methoxy-4-(4-(2-nitro-5-vinylphenoxy)phenethyl)-[1,1'-biphenyl]-2-ol (17)

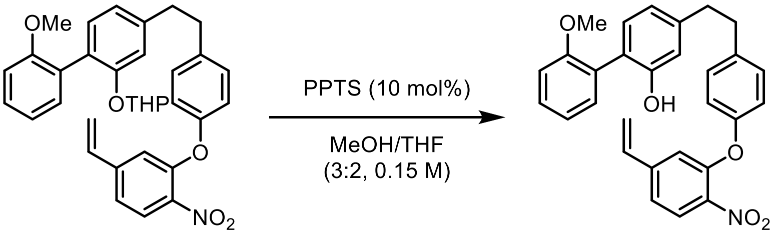

16 **17**

A solution of **16** (154 mg, 0.28 mmol, 1.0 equiv) and PPTS (19 mg, 10 mol%) in MeOH (1.1 mL) and THF (0.70 mL) was stirred at rt under air atmosphere for 17 h. The reaction mixture was concentrated under reduced pressure, and the resulting residue was purified by flash column chromatography (AcOEt/hexane 15:85 to 20:80) to afford 126 mg (97%) of **17** as a white solid. **R_f**: 0.30 (AcOEt/hexane 25:75, red in p-anisaldehyde). **^{1}H NMR** (500 MHz, $CDCl_3$) δ: 7.95 (d, *J* = 8.6 Hz, 1H), 7.39 (ddd, *J* = 8.3, 7.3, 1.7 Hz, 1H), 7.35 (dd, *J* = 7.6, 1.8 Hz, 1H), 7.25 – 7.22 (m, 2H), 7.20 (dd, *J* = 8.4, 1.8 Hz, 1H), 7.19 (d, *J* = 7.8 Hz, 1H), 7.12 (td, *J* = 7.5, 1.2 Hz, 1H), 7.06 (dd, *J* = 8.3, 1.2 Hz, 1H), 7.02 – 6.98 (m, 2H), 6.98 (d, *J* = 1.8 Hz, 1H), 6.90 (d, *J* = 1.8 Hz, 1H), 6.85 (dd, *J* = 7.8, 1.8 Hz, 1H), 6.61 (dd, *J* = 17.5, 10.9 Hz, 1H), 6.28 (s, 1H), 5.77 (d, *J* = 17.6 Hz, 1H), 5.41 (d, *J* = 10.9 Hz, 1H), 3.91 (s, 3H), 3.01 – 2.92 (m, 4H). **^{13}C NMR** (126 MHz, $CDCl_3$) δ: 155.6 (C), 153.9 (C), 153.8 (C), 151.7 (C), 144.0 (C), 143.2 (C), 140.1 (C), 138.4 (C), 134.9 (CH), 132.6 (CH), 131.4 (CH), 130.2 (2xCH), 129.3 (CH), 127.2 (C), 126.4 (CH), 124.1 (C), 122.4 (CH), 121.4 (CH), 120.4 (CH), 119.4 (2xCH), 118.7 (CH_2), 117.9 (CH), 117.5 (CH), 111.8 (CH), 56.4 (OCH_3), 37.6 (CH_2), 37.0 (CH_2). **HRMS** (APCI+) *m/z* calcd. for $C_{29}H_{26}NO_5$ [M+H]: 468.1805; found: 468.1806.

(E)-12-nitro-7,8-dehydroisoriccardin C 3-methyl ether (18)

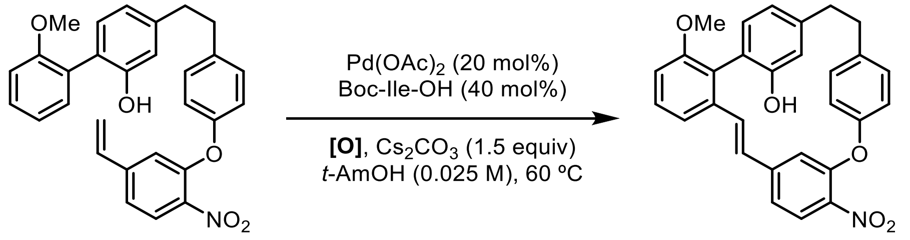

17 **18**

Table S4. Optimization for the cyclization of **17**.

Entry[a]	[O] (equiv)	Atm	t (h)	**18** yield (%)	**17** recov. (%)
1	$Cu(OAc)_2 \cdot H_2O$ (1.0)	air	6.25	32	26
2	$Cu(OAc)_2$ (1.0)	O_2	17	23	0
**3**	**$Cu(OAc)_2$ (0.20)**	**O_2**	**17**	**57**	**Traces**
5	$Cu(OAc)_2$ (0.10)	O_2	26	34	40
6[b]	$Cu(OAc)_2$ (0.20)	O_2	24	27	41
7	-	O_2	20	25	35

[a] Reactions performed at 0.10 mmol scale of **17** in an oven dried sealed tube. Yields are isolated.
[b] $Pd(OAc)_2$ (10 mol%) and Boc-Ile-OH (20 mol%).

Optimized procedure in entry 3

A mixture of **17** (46.8 mg, 0.10 mmol, 1.0 equiv), $Pd(OAc)_2$ (4.5 mg, 20 mol%), Boc-Ile-OH (9.3 mg, 40 mol%), $Cu(OAc)_2$ (3.6 mg, 20 mol%) and Cs_2CO_3 (49 mg, 1.5 equiv) in *t*-AmOH (4.0 mL) was stirred in a sealed tube under oxygen atmosphere at 60 ºC (oil bath) for 17 h. After cooling to rt, the reaction mixture was diluted with AcOEt and filtered through a Florisil pad, washing the solids with AcOEt for several times. The filtrate was concentrated under reduced pressure, and the crude residue was purified by flash column chromatography (AcOEt/hexane 20:80) to afford 26.5 mg (57%) of **18** as a bright yellow solid. **R_f**: 0.30 (AcOEt/hexane 30:70, orange in p-anisaldehyde). **^{1}H NMR** (500 MHz, $CDCl_3$) δ: 7.94 (d, *J* = 8.4 Hz, 1H), 7.44 (dd, *J* = 8.0, 1.2 Hz, 1H), 7.38 (t, *J* = 8.0 Hz, 1H), 7.29 (dd, *J* = 8.3, 2.2 Hz, 1H), 7.06 (dd, *J* = 8.3, 2.3 Hz, 1H), 7.00 (dd, *J* = 8.3, 2.5 Hz, 1H), 6.96 (dd, *J* = 8.1, 1.1 Hz, 1H), 6.93 – 6.87 (m, 3H), 6.83 (d, *J* = 1.7 Hz, 1H), 6.73 (d, *J* = 7.6 Hz, 1H), 6.54 (dd, *J* = 7.7, 1.7 Hz, 1H), 6.44 (d, *J* = 3.3 Hz, 1H), 6.42 (d, *J* = 11.1 Hz, 1H), 5.02 (s, 1H), 3.80 (s, 3H), 3.29 – 3.16 (m, 2H), 3.10 – 3.01 (m, 2H). **^{13}C NMR** (126 MHz, $CDCl_3$) δ: 157.3 (C), 154.3 (C), 153.3 (C), 151.7 (C), 143.7 (C), 142.2 (C), 138.3 (C), 137.8 (C), 137.1 (C), 131.9 (CH), 131.4 (CH), 131.4 (CH), 130.7 (CH),

129.5 (CH), 126.8 (CH), 126.2 (CH), 125.3 (C), 122.1 (CH), 121.9 (CH), 121.7 (CH), 121.5 (CH), 119.9 (C), 118.3 (CH), 116.8 (CH), 111.5 (CH), 111.1 (CH), 56.2 (OCH_3), 35.7 (CH_2), 35.1 (CH_2). **HRMS** (APCI+) *m/z* calcd. for $C_{29}H_{24}NO_5$ [M+H]: 466.1649; found: 466.1643.

Crystallization of compound **18** from CH_2Cl_2/heptane afforded suitable crystals for X-ray diffraction analysis. The structure was deposited in the Cambridge Structural Database: 2526903.

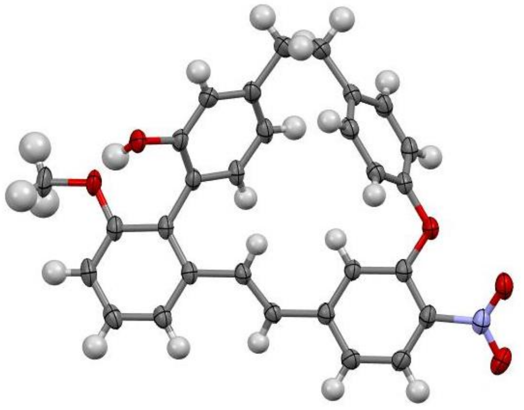

Figure S3. Thermal ellipsoid plot of compound **18** with ellipsoid contour 50% probability levels.

Table S5. Crystal parameters for compound **18**.

M_r	465.48
Crystal system, space group	Triclinic, *P*-1
Temperature (K)	100
a, *b*, *c* (Å)	8.4197 (2), 11.2311 (3), 12.8371 (3)
α, *β*, *γ* (°)	98.904 (2), 99.657 (2), 106.016 (3)
V ($Å^3$)	1124.07 (6)
Z	2
Radiation type	Cu *Kα*
μ (mm^{-1})	0.77
Crystal size (mm)	0.11 × 0.09 × 0.01

5. NMR Spectra

4-iodo-3-((tetrahydropyran-2-yl)oxy)benzaldehyde (1)

^{1}H NMR (500 MHz, $CDCl_3$)

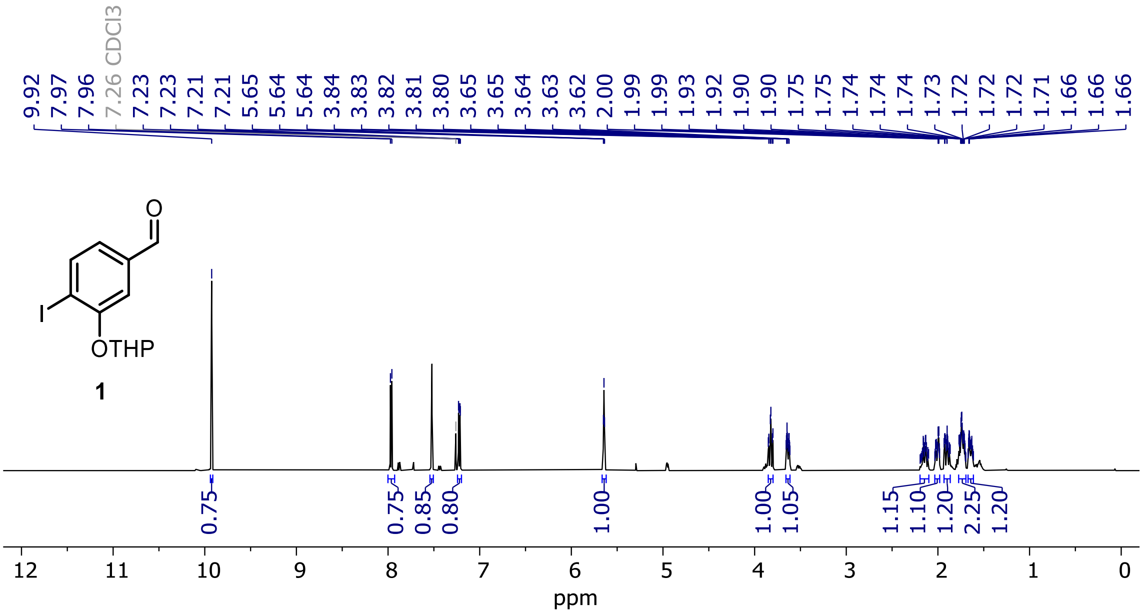

DEPT-135

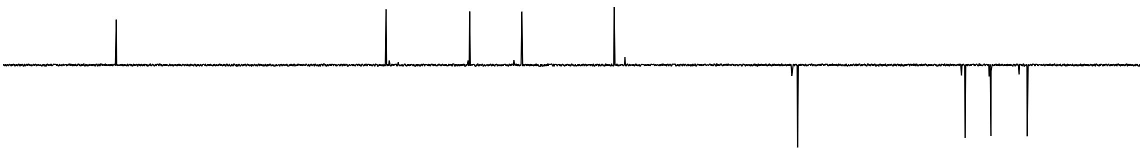

^{13}C NMR (126 MHz, $CDCl_3$)

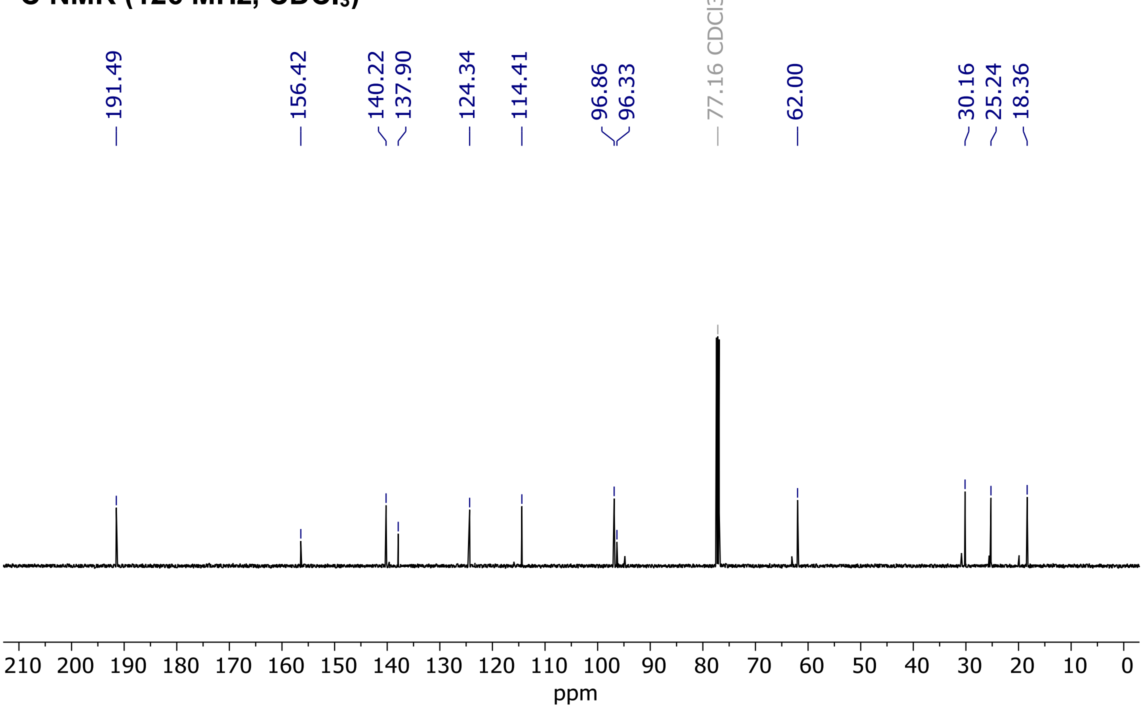

2'-methoxy-2-((tetrahydropyran-2-yl)oxy)-[1,1'-biphenyl]-4-carbaldehyde (2)

^{1}H NMR (300 MHz, $CDCl_3$)

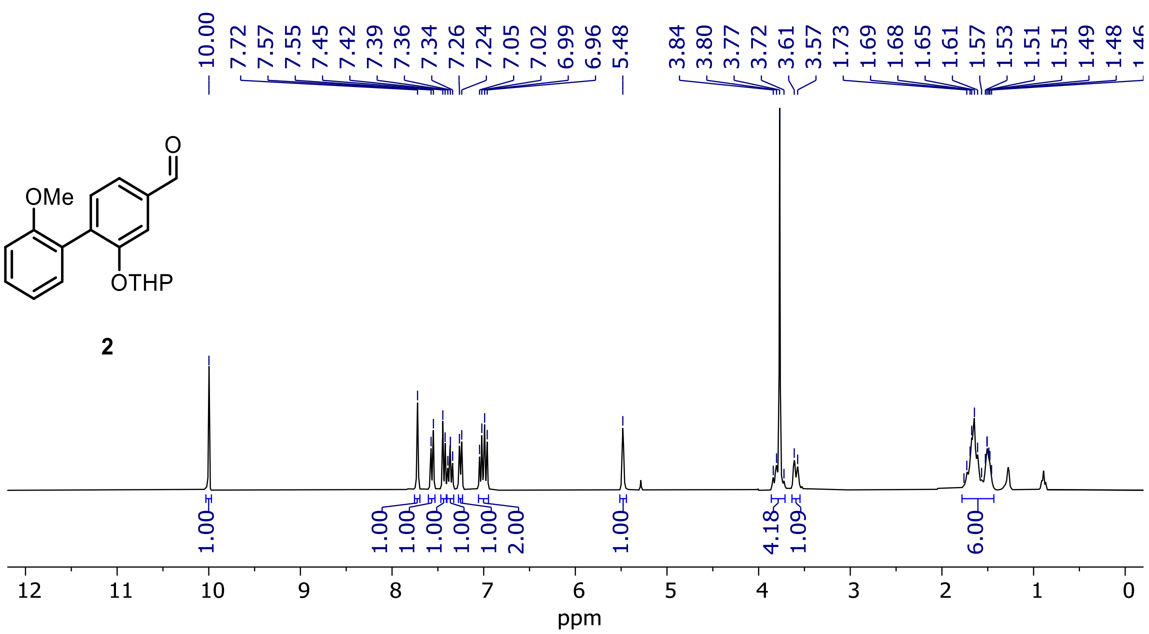

DEPT-135

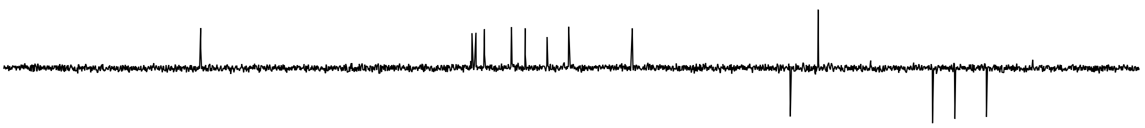

^{13}C NMR (75 MHz, $CDCl_3$)

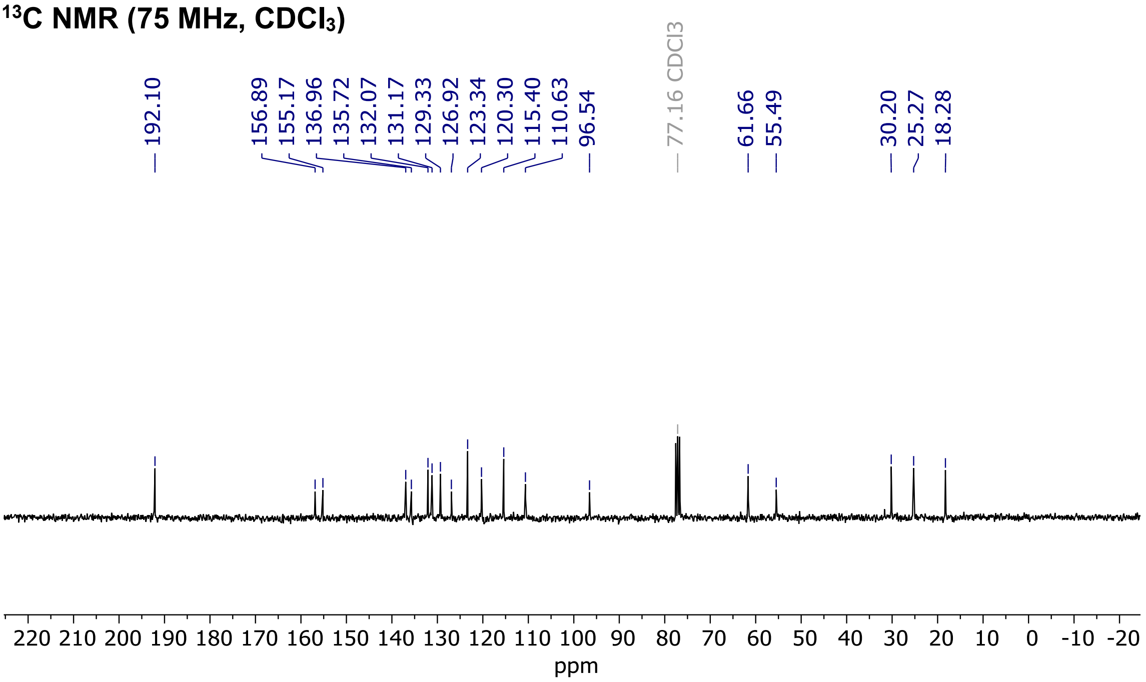

2-((4-(4-bromophenethyl)-2'-methoxy-[1,1'-biphenyl]-2-yl)oxy)tetrahydropyran (4)

^{1}H NMR (500 MHz, $CDCl_3$)

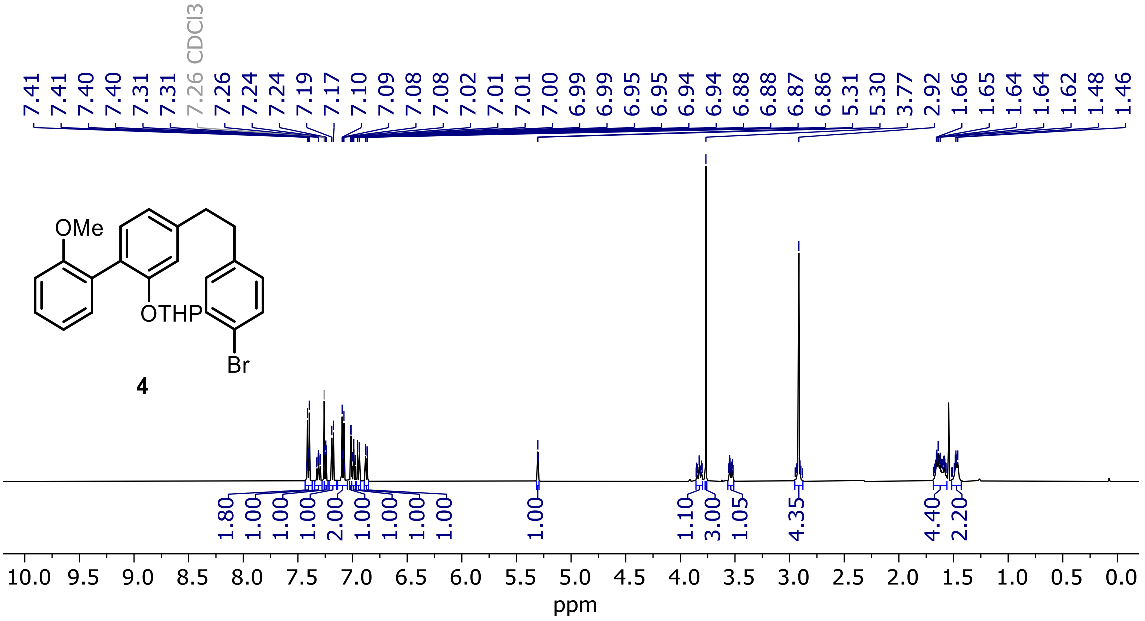

DEPT-135

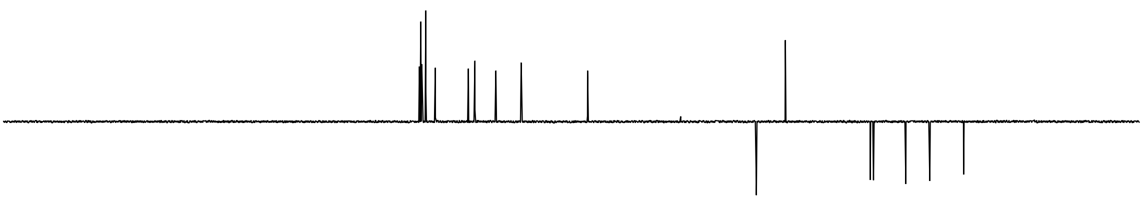

^{13}C NMR (126 MHz, $CDCl_3$)

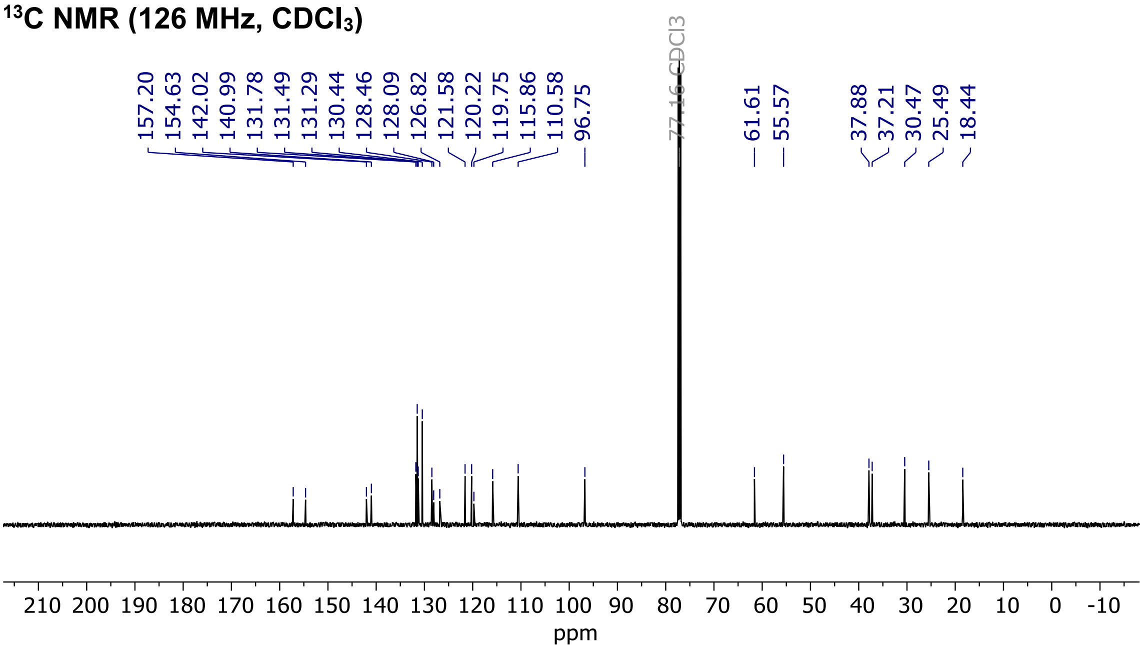

2-((2'-methoxy-4-(4-(2-methoxy-5-vinylphenoxy)phenethyl)-[1,1'-biphenyl]-2-yl)oxy)tetrahydropyran (5)

^{1}H NMR (500 MHz, $CDCl_3$)

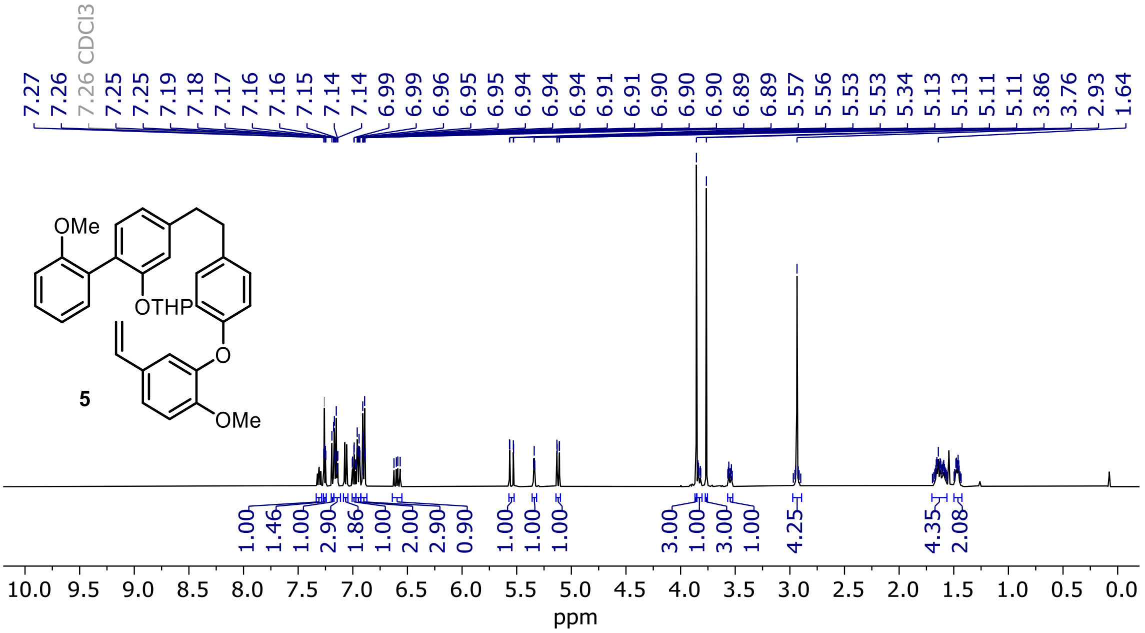

DEPT-135

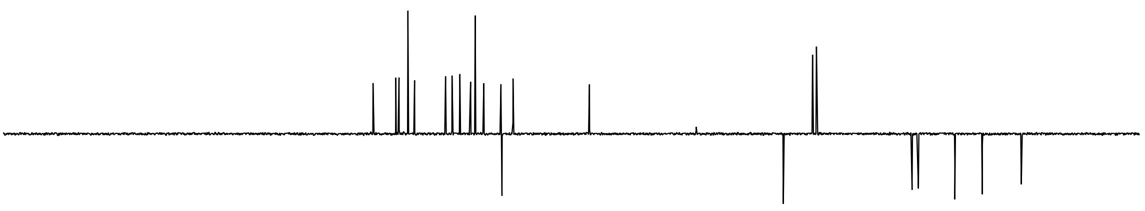

^{13}C NMR (126 MHz, $CDCl_3$)

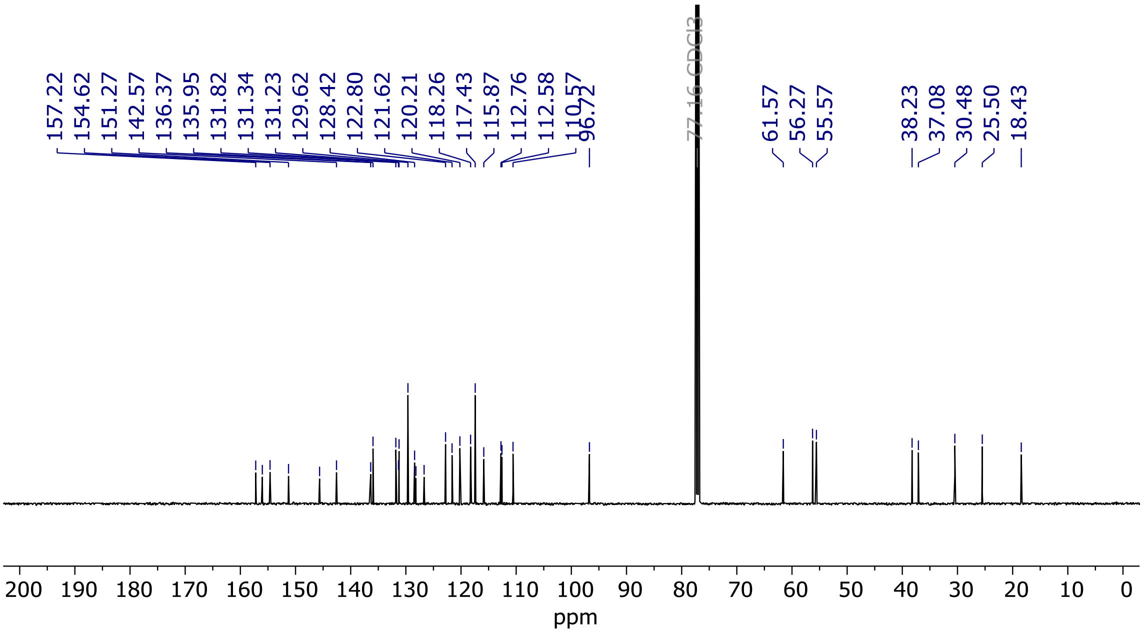

2'-methoxy-4-(4-(2-methoxy-5-vinylphenoxy)phenethyl)-[1,1'-biphenyl]-2-ol (6)

^{1}H NMR (300 MHz, $CDCl_3$)

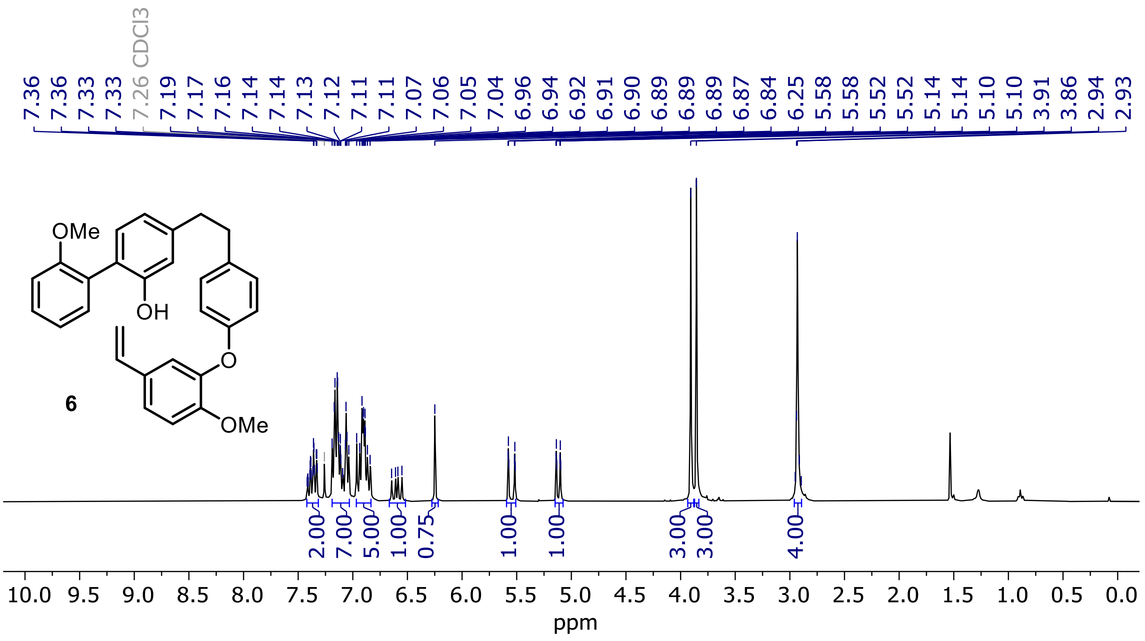

DEPT-135

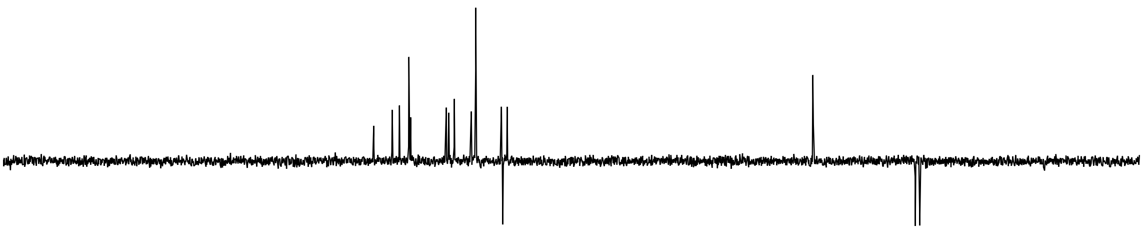

^{13}C NMR (75 MHz, $CDCl_3$)

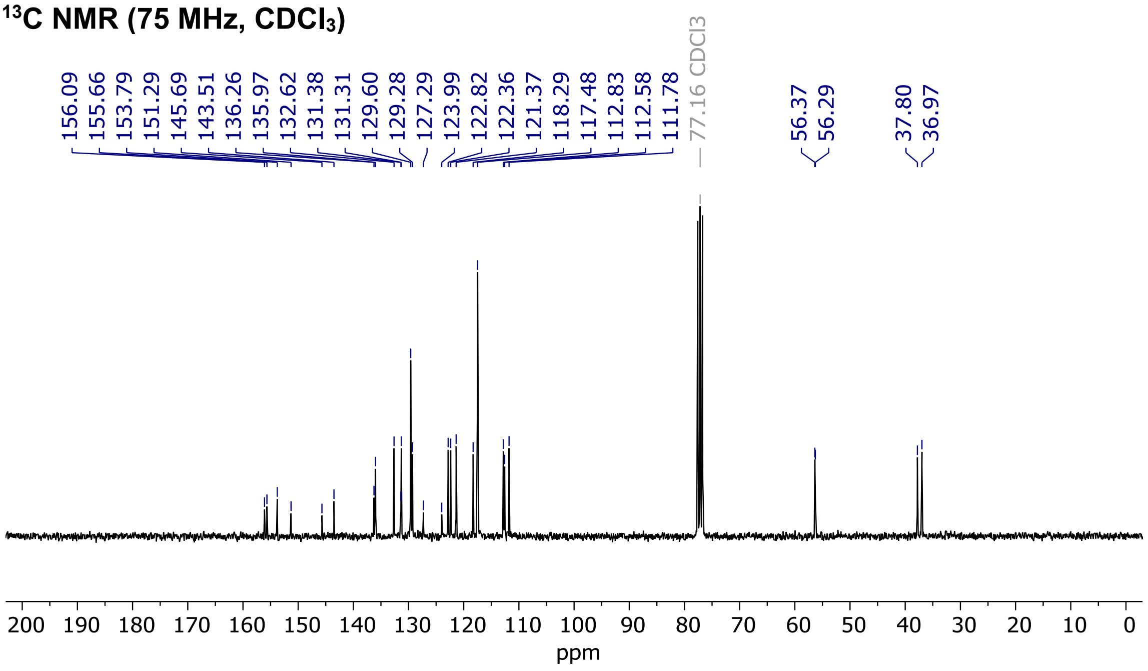

7,8-dehydroisoriccardin C 3,12-dimethyl ether (7)

^{1}H NMR (500 MHz, $CDCl_3$)

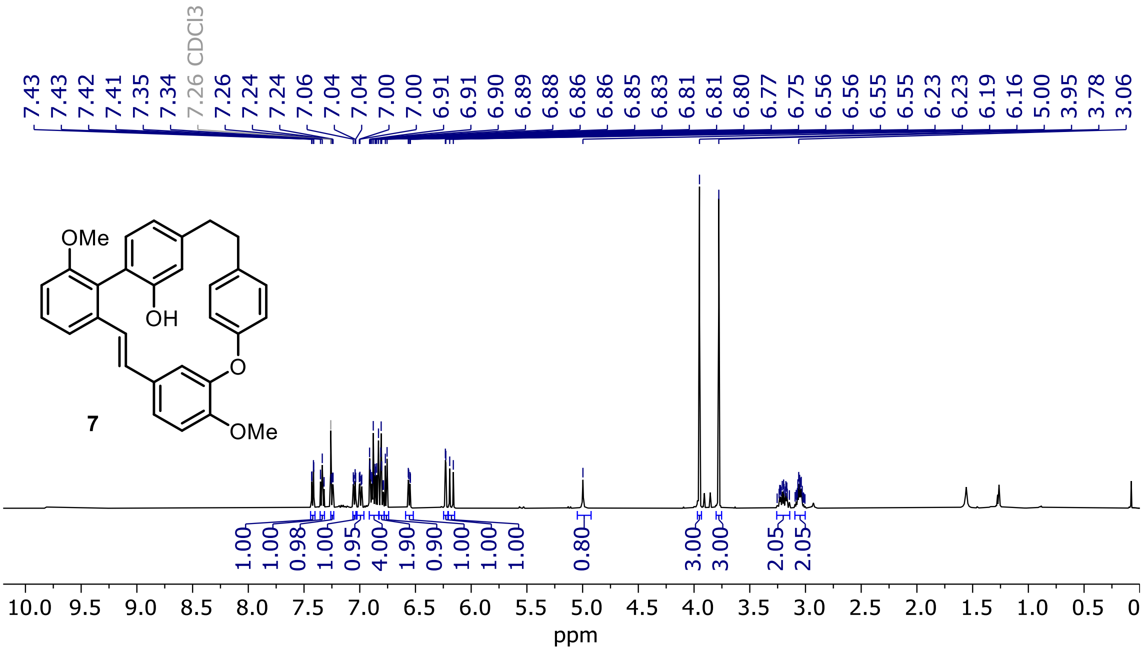

DEPT-135

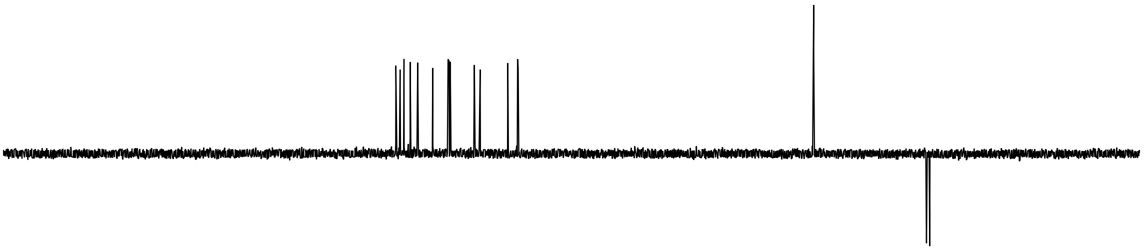

^{13}C NMR (126 MHz, $CDCl_3$)

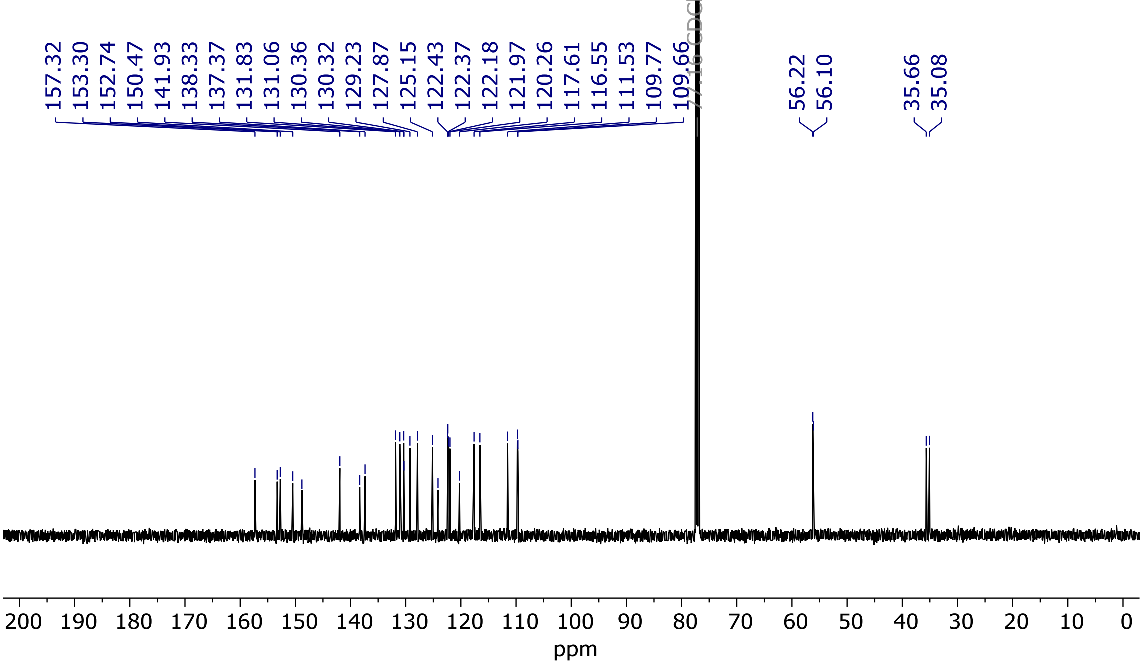

Isoriccardin C (8)

^{1}H NMR (500 MHz, $CDCl_3$)

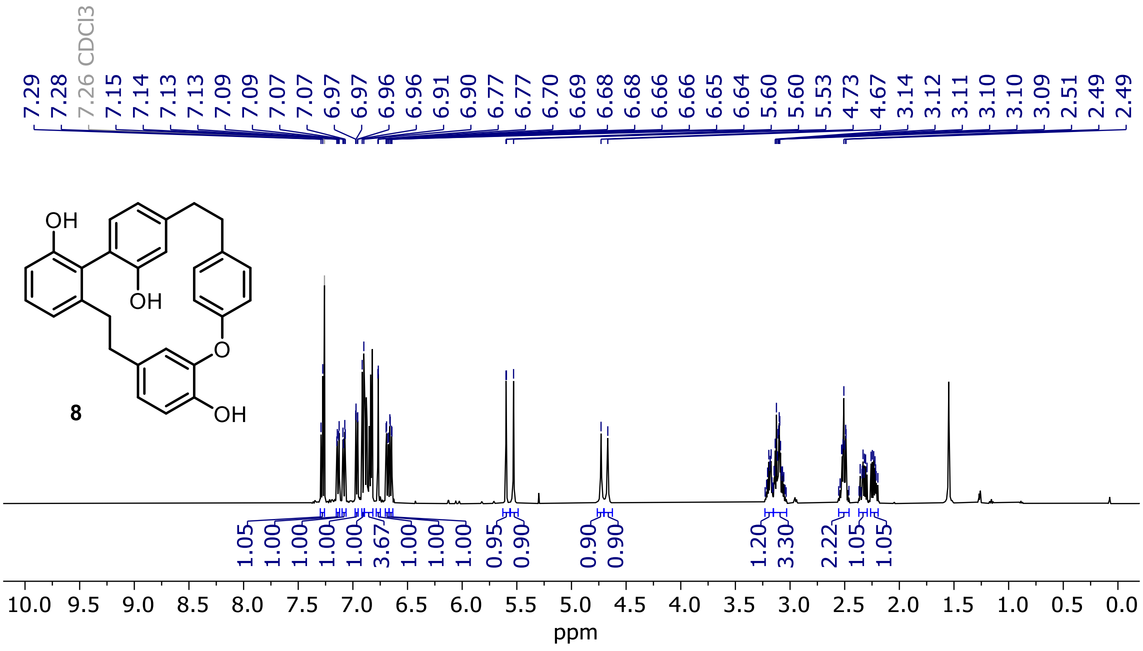

DEPT-135

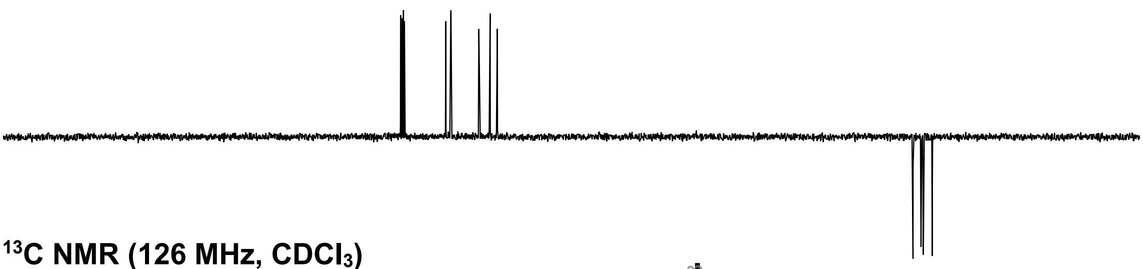

^{13}C NMR (126 MHz, $CDCl_3$)

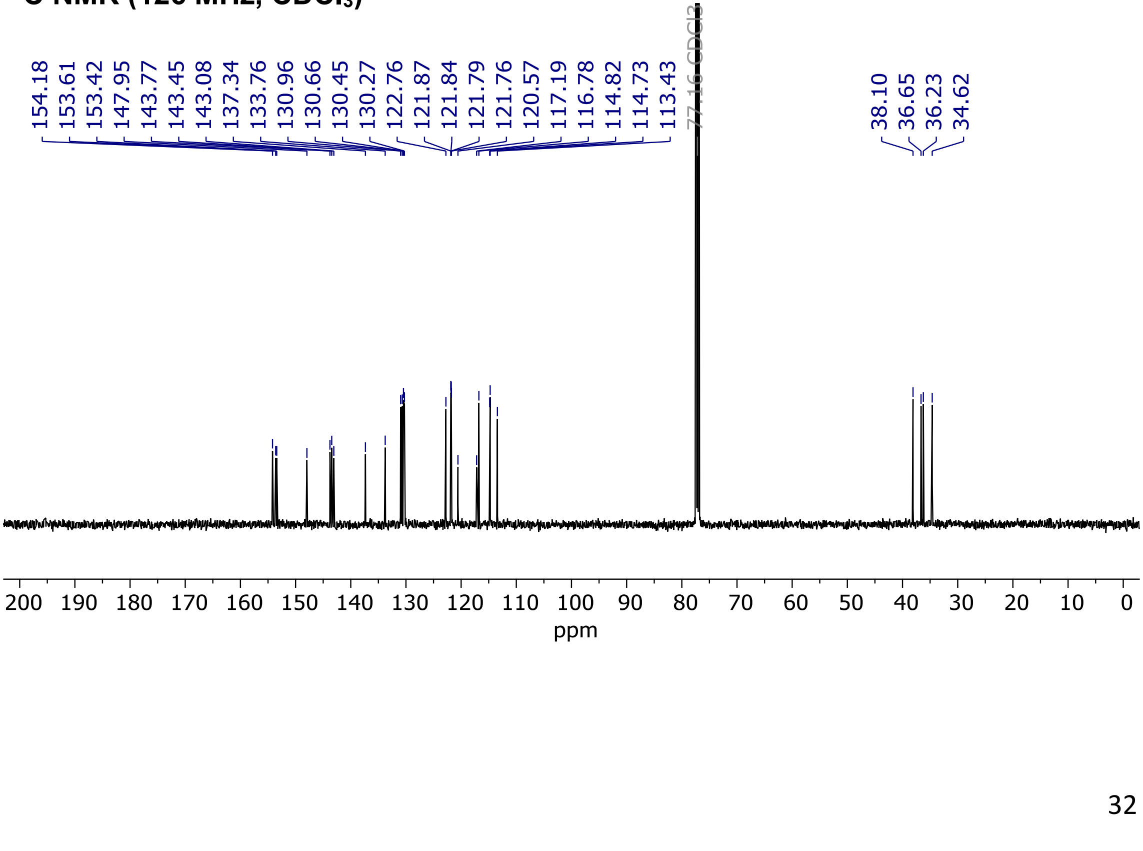

4'-methoxy-2-((tetrahydropyran-2-yl)oxy)-[1,1'-biphenyl]-4-carbaldehyde (9)

^{1}H NMR (500 MHz, $CDCl_3$)

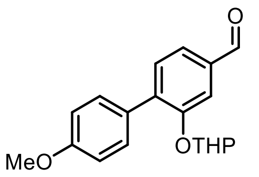

9

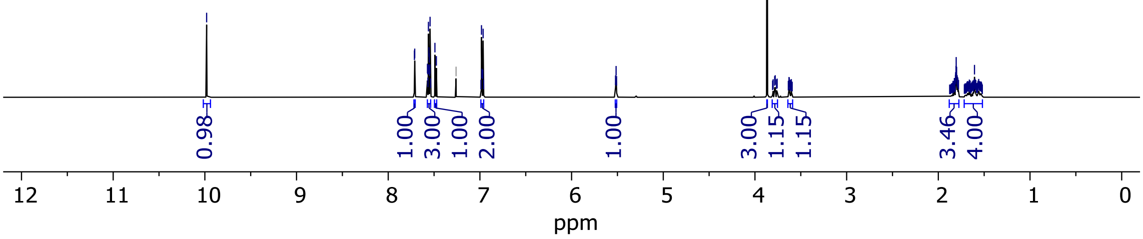

DEPT-135

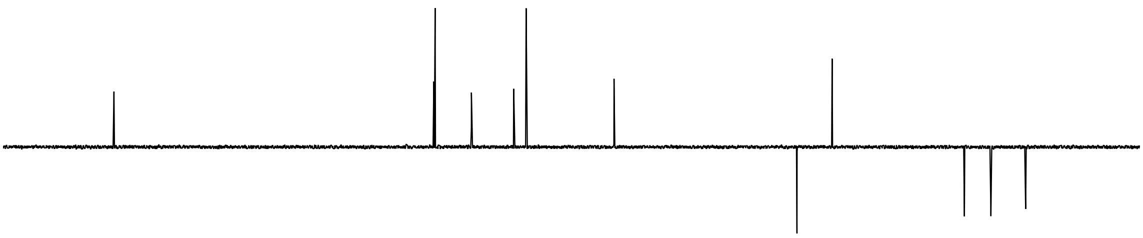

^{13}C NMR (126 MHz, $CDCl_3$)

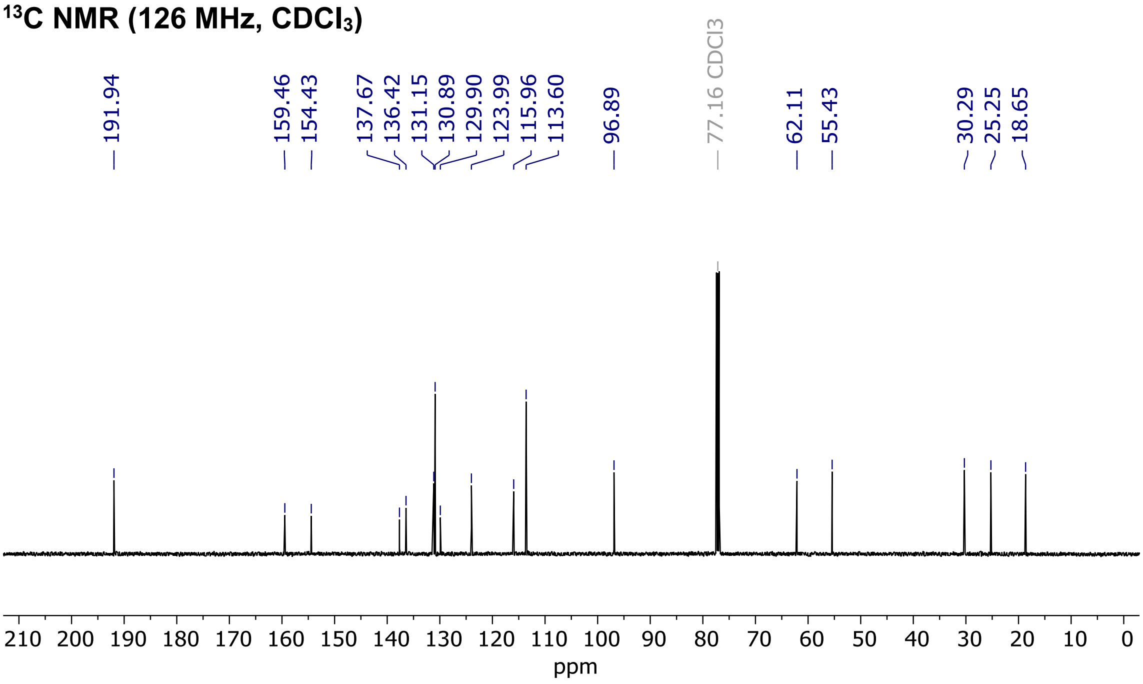

2-((4-(4-bromophenethyl)-4'-methoxy-[1,1'-biphenyl]-2-yl)oxy)tetrahydropyran (10)

^{1}H NMR (500 MHz, $CDCl_3$)

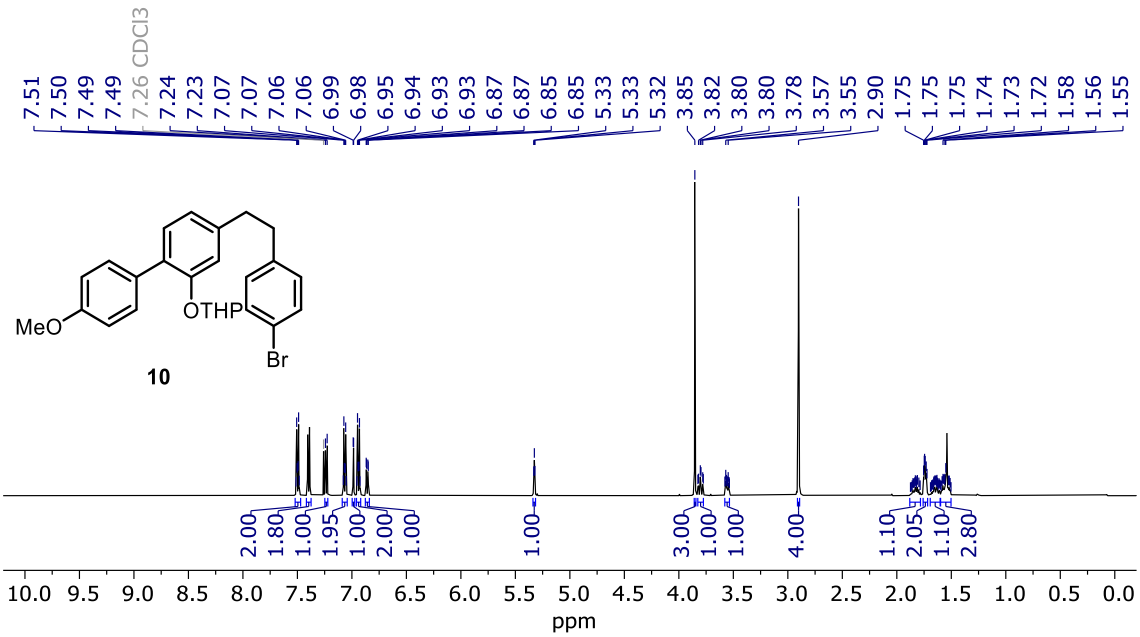

DEPT-135

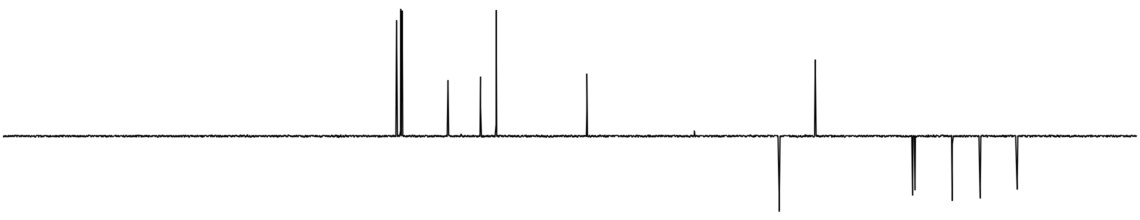

^{13}C NMR (126 MHz, $CDCl_3$)

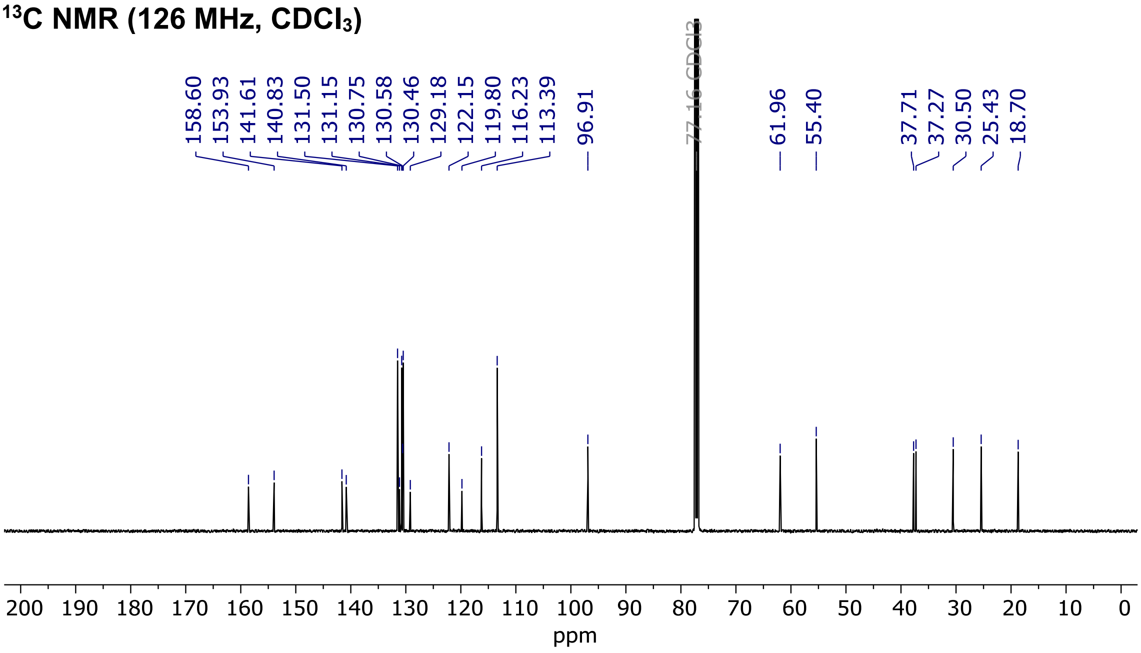

2-((4'-methoxy-4-(4-(2-methoxy-5-vinylphenoxy)phenethyl)-[1,1'-biphenyl]-2-yl) oxy)tetrahydropyran (11)

^{1}H NMR (500 MHz, $CDCl_3$)

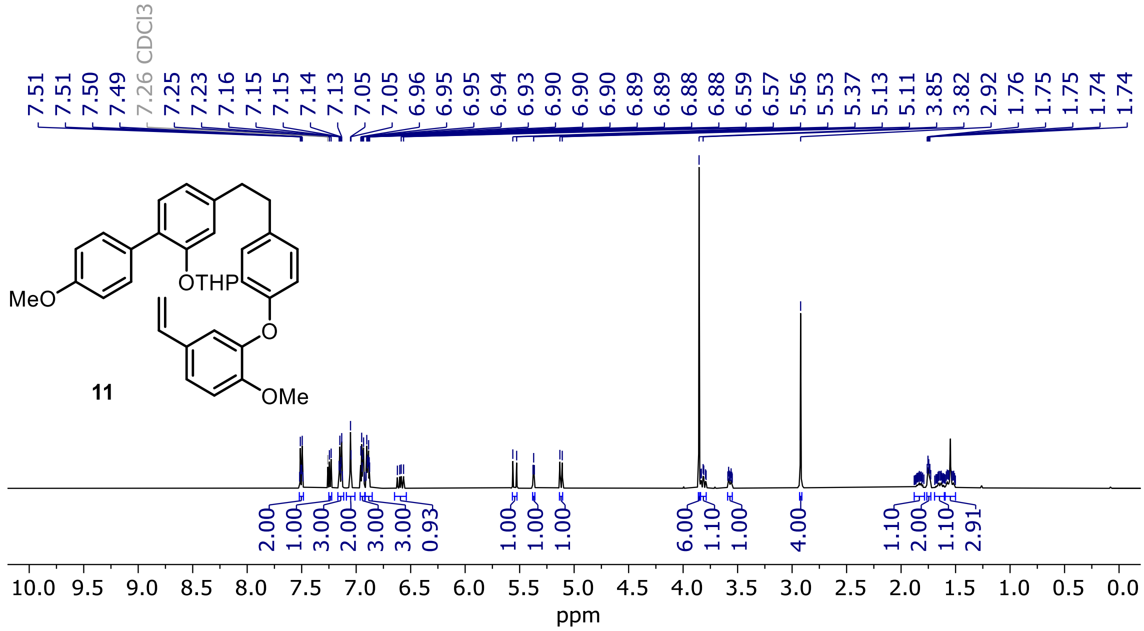

DEPT-135

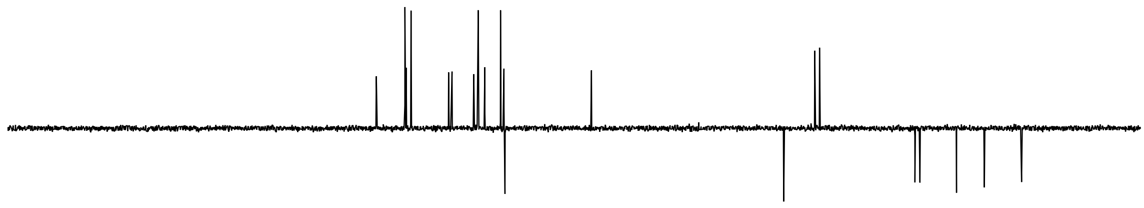

^{13}C NMR (126 MHz, $CDCl_3$)

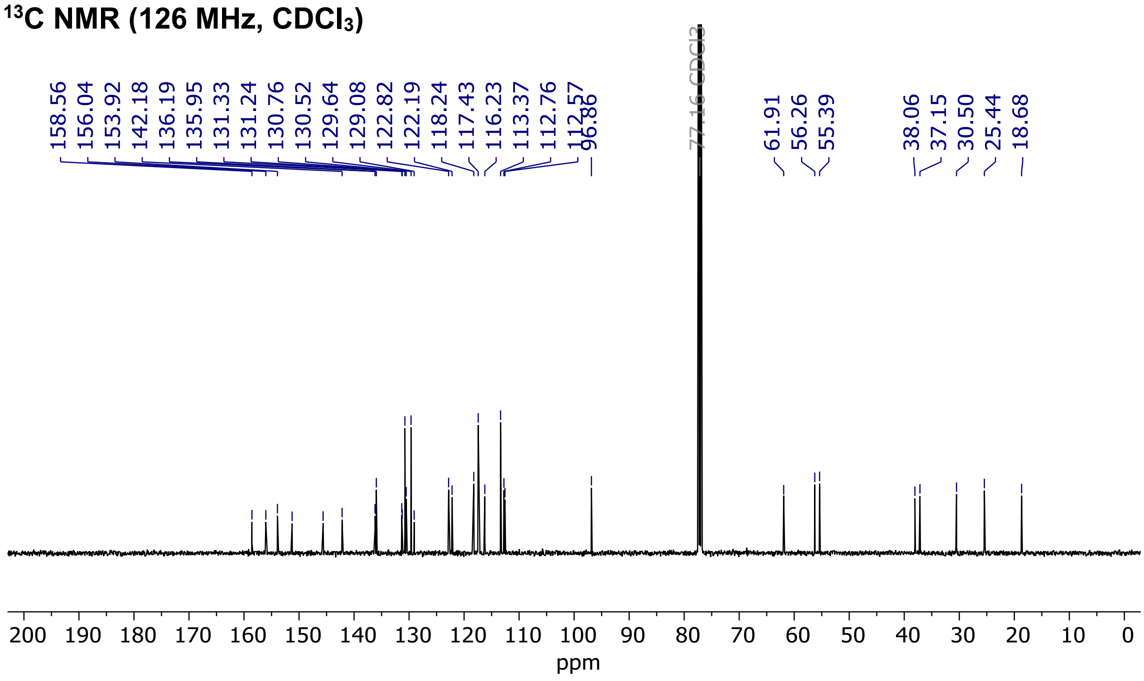

4'-methoxy-4-(4-(2-methoxy-5-vinylphenoxy)phenethyl)-[1,1'-biphenyl]-2-ol (12)

^{1}H NMR (500 MHz, $CDCl_3$)

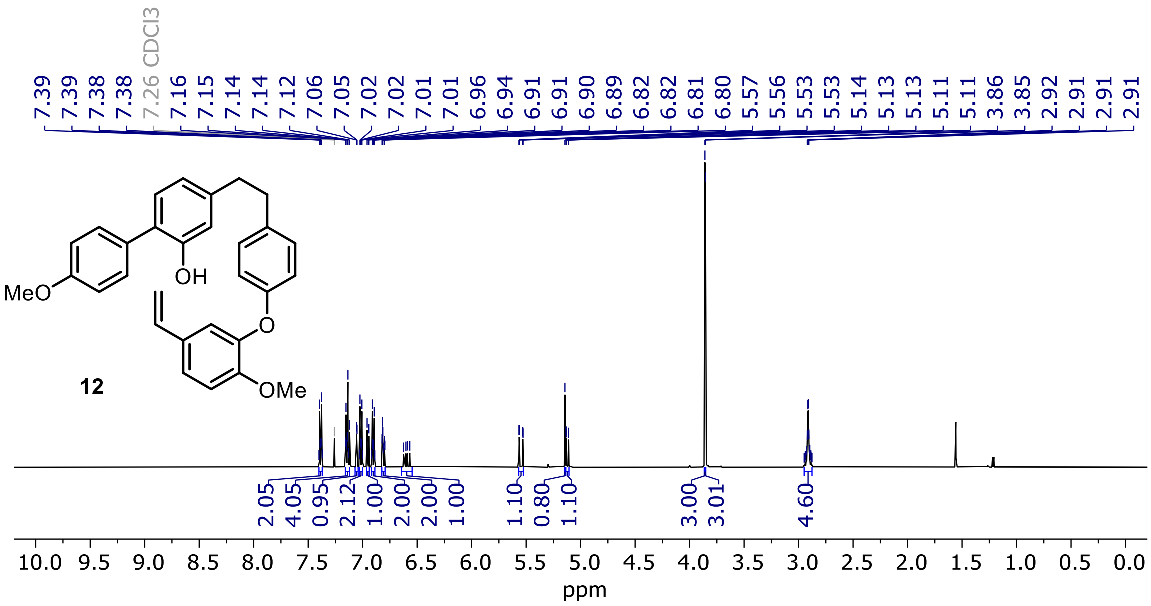

DEPT-135

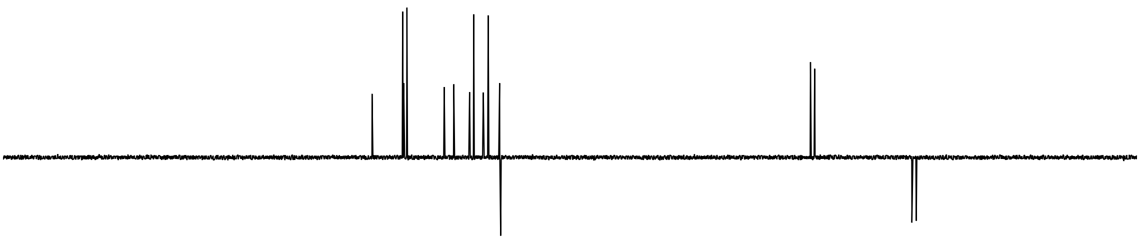

^{13}C NMR (126 MHz, $CDCl_3$)

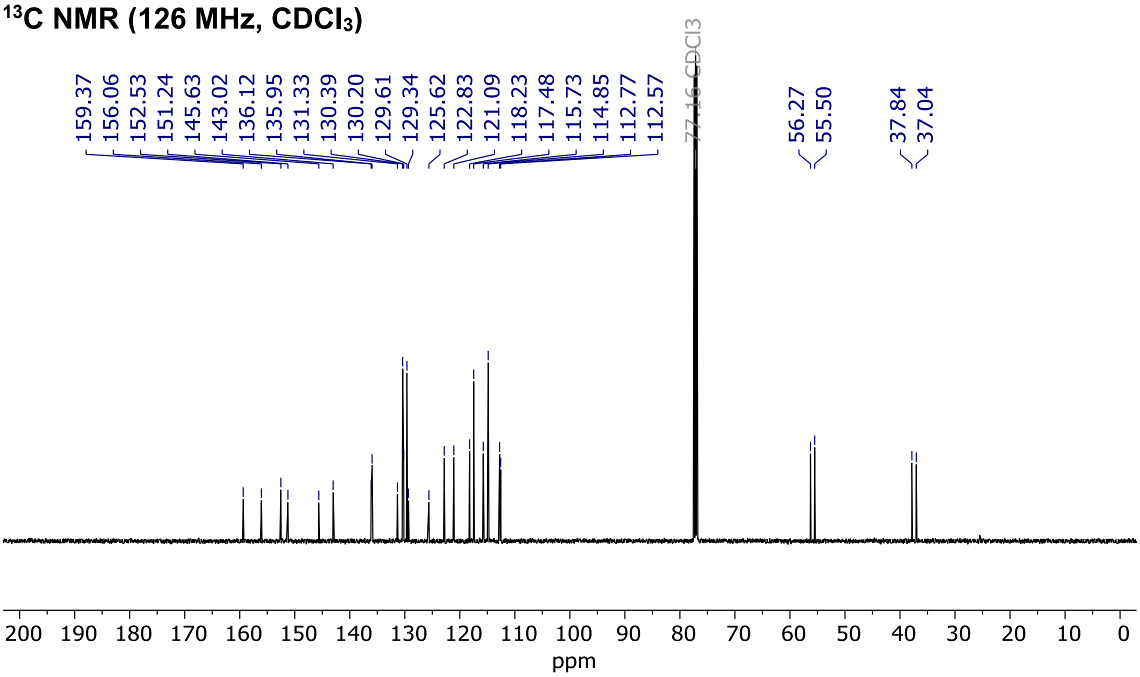

(E)-7,8-dehydroisoriccardin D 3,12-dimethyl ether (13)

^{1}H NMR (500 MHz, CDCl$_3$)

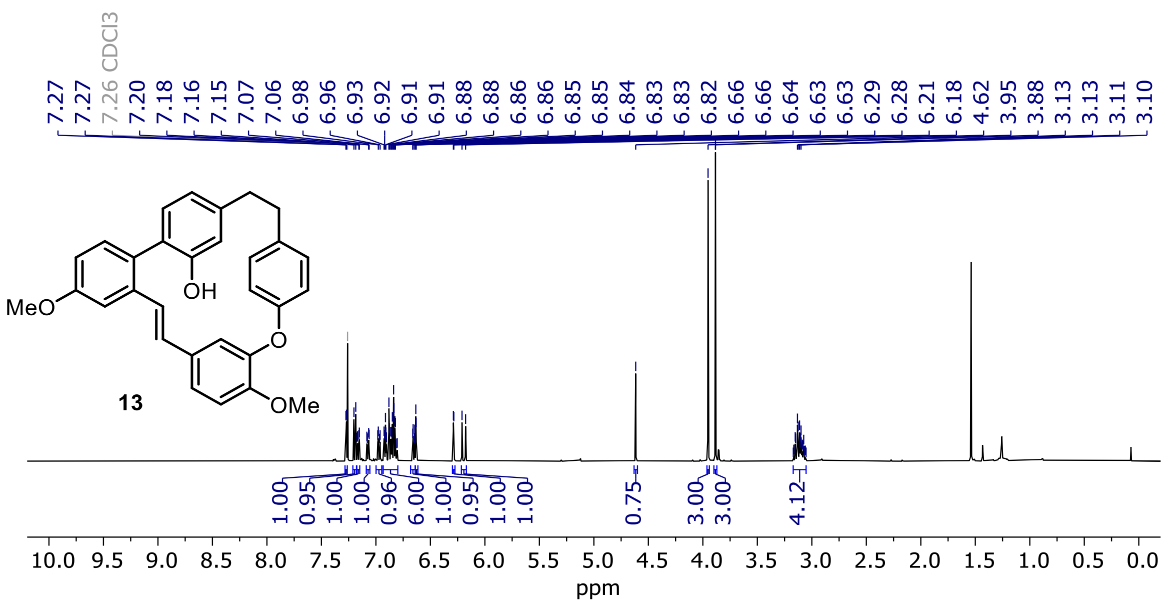

13

DEPT-135

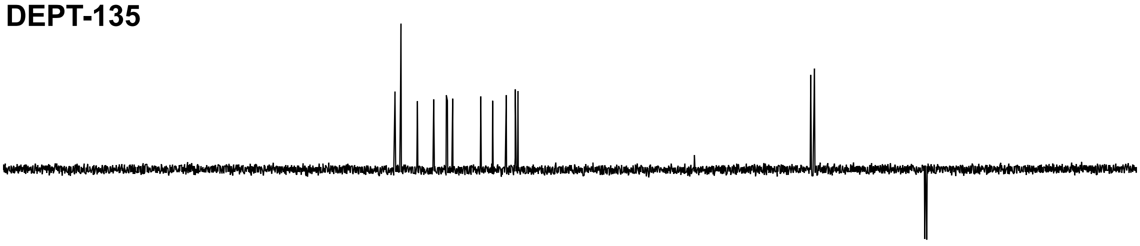

^{13}C NMR (126 MHz, CDCl$_3$)

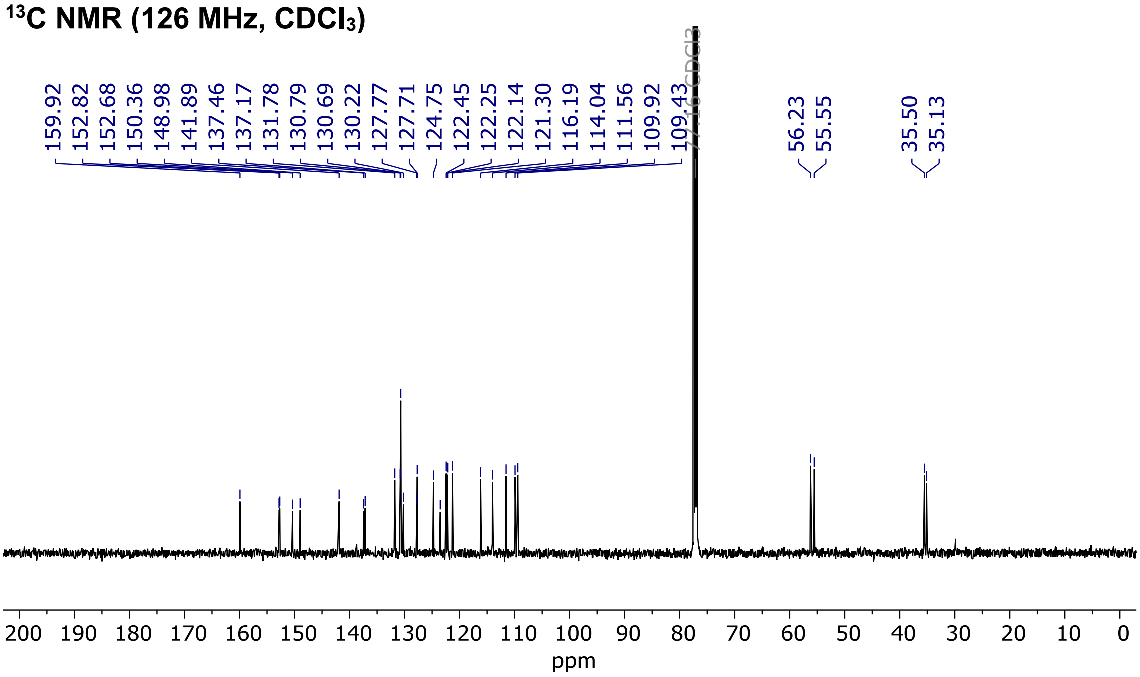

Isoriccardin D (14)

^{1}H NMR (500 MHz, DMSO-d_6)

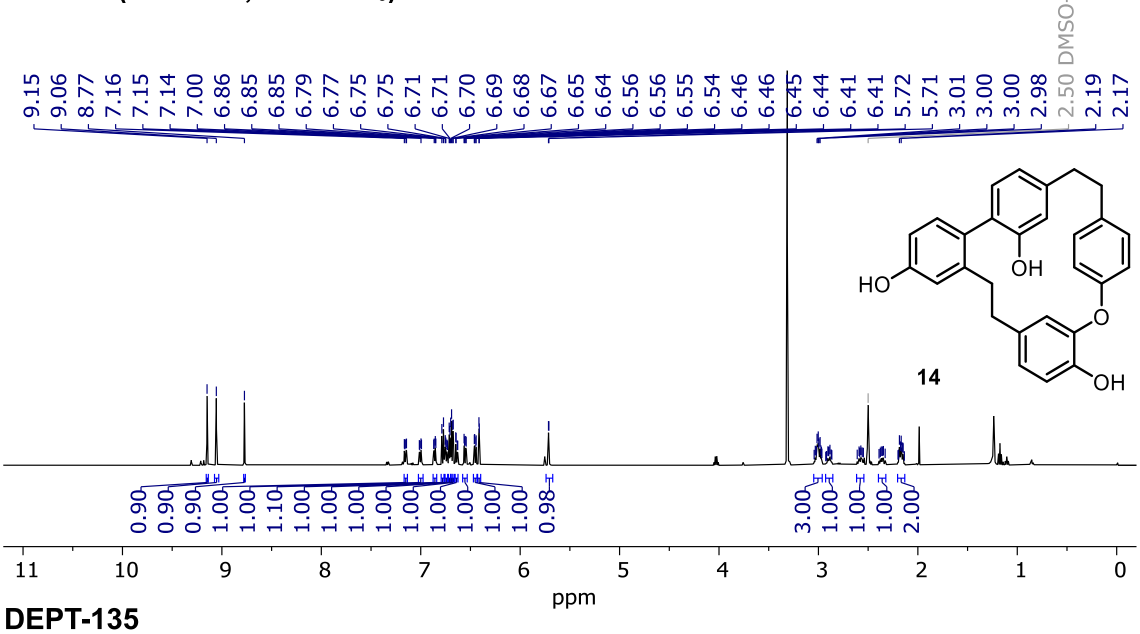

DEPT-135

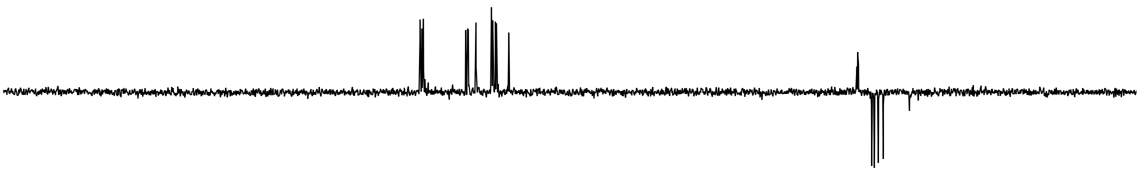

^{13}C NMR (126 MHz, DMSO-d_6)

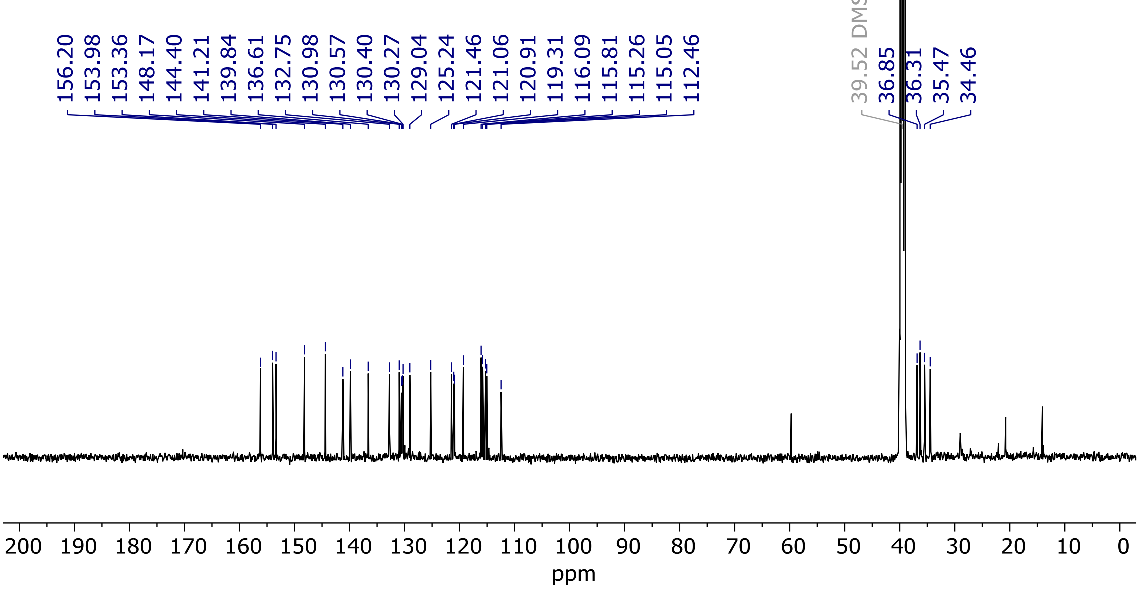

4-(2-(2'-methoxy-2-((tetrahydropyran-2-yl)oxy)-[1,1'-biphenyl]-4-yl)ethyl) phenol (15)

^{1}H NMR (500 MHz, CD_2Cl_2)

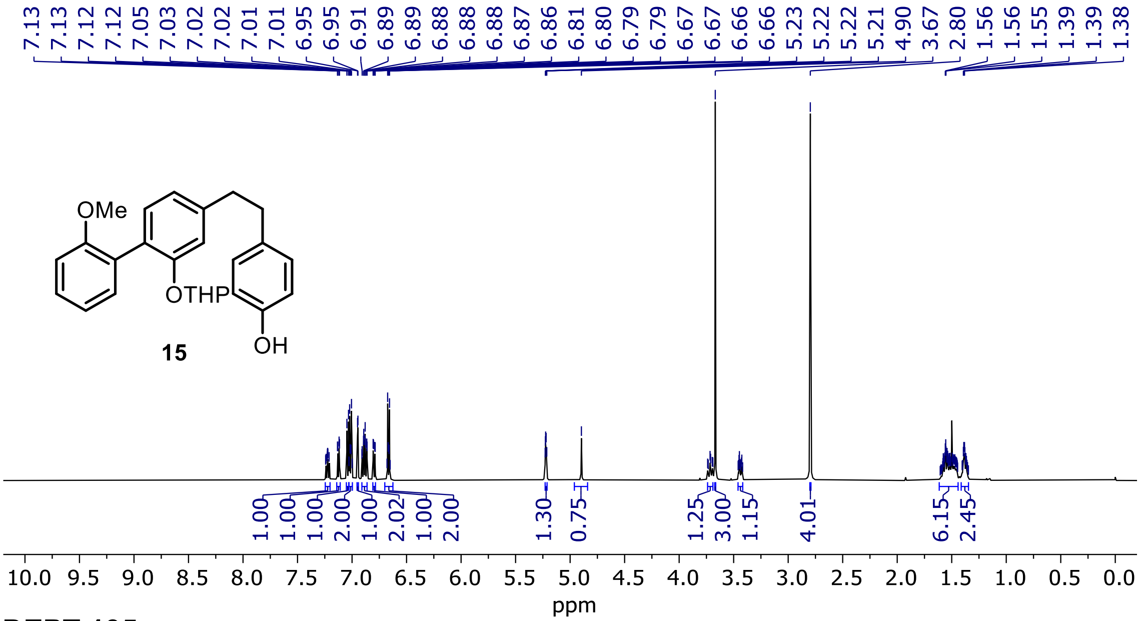

DEPT-135

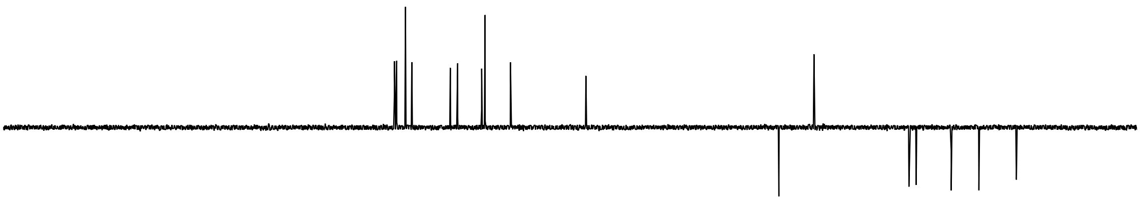

^{13}C NMR (126 MHz, CD_2Cl_2)

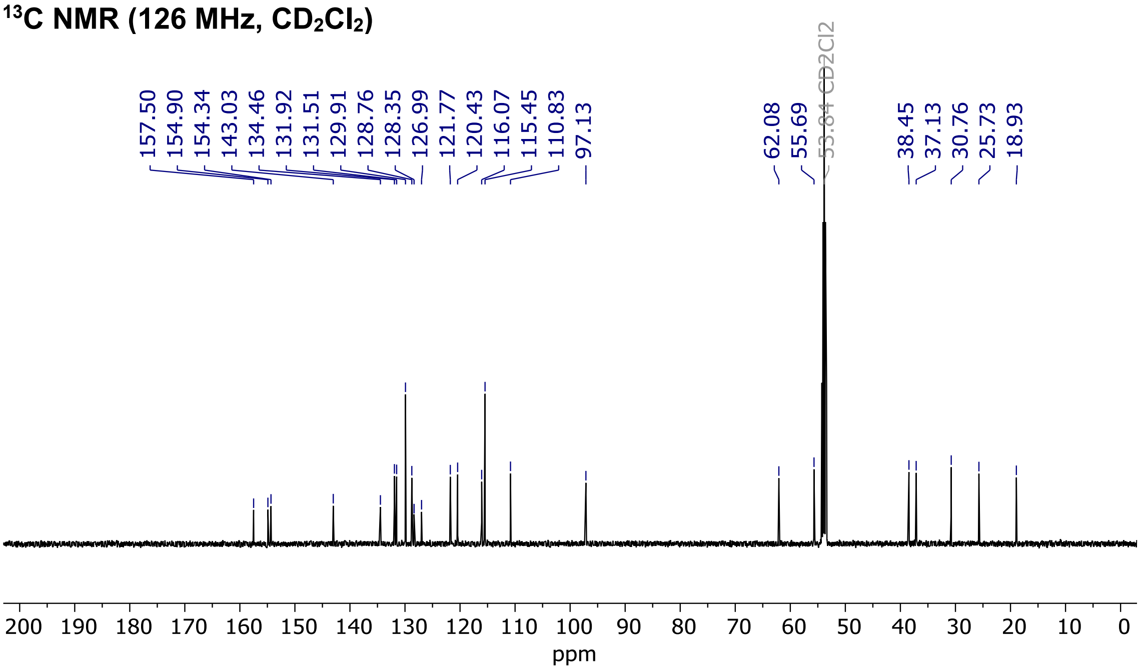

3-fluoro-4-nitrostyrene (S4)

^1H NMR (500 MHz, $CDCl_3$)

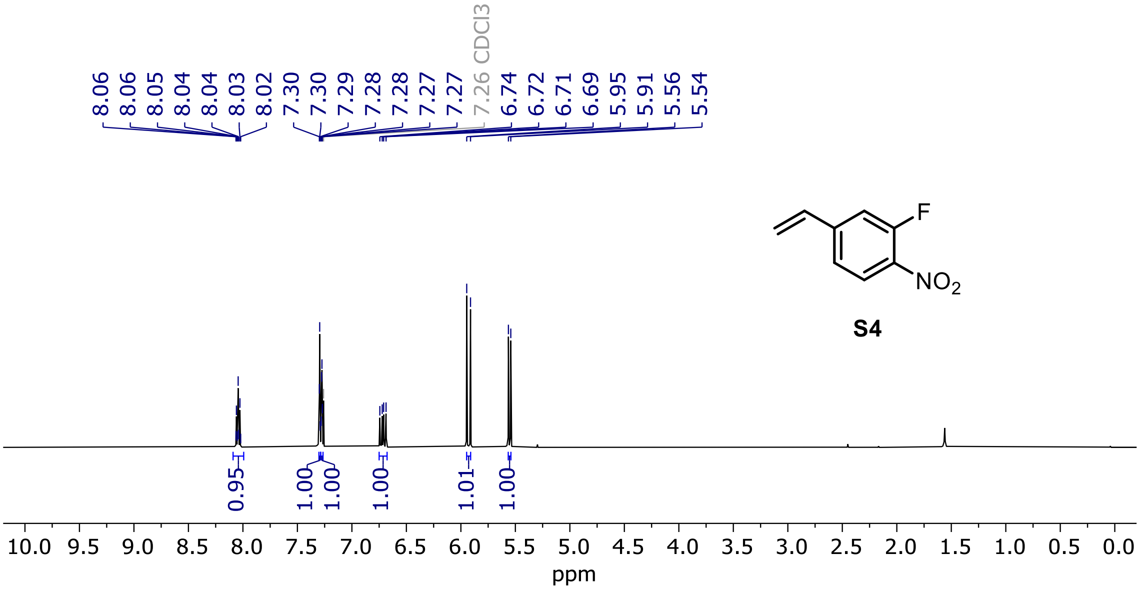

S4

DEPT-135

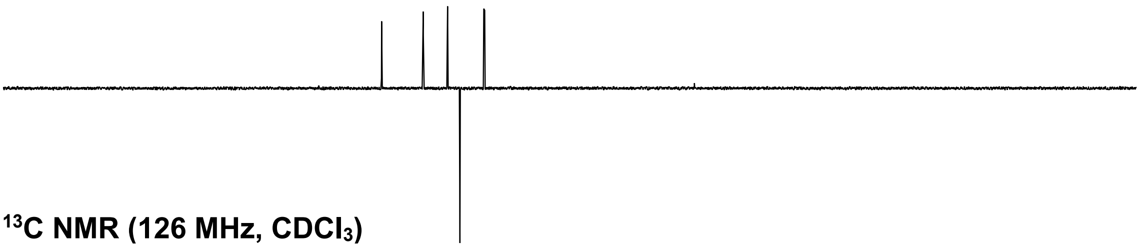

^{13}C NMR (126 MHz, $CDCl_3$)

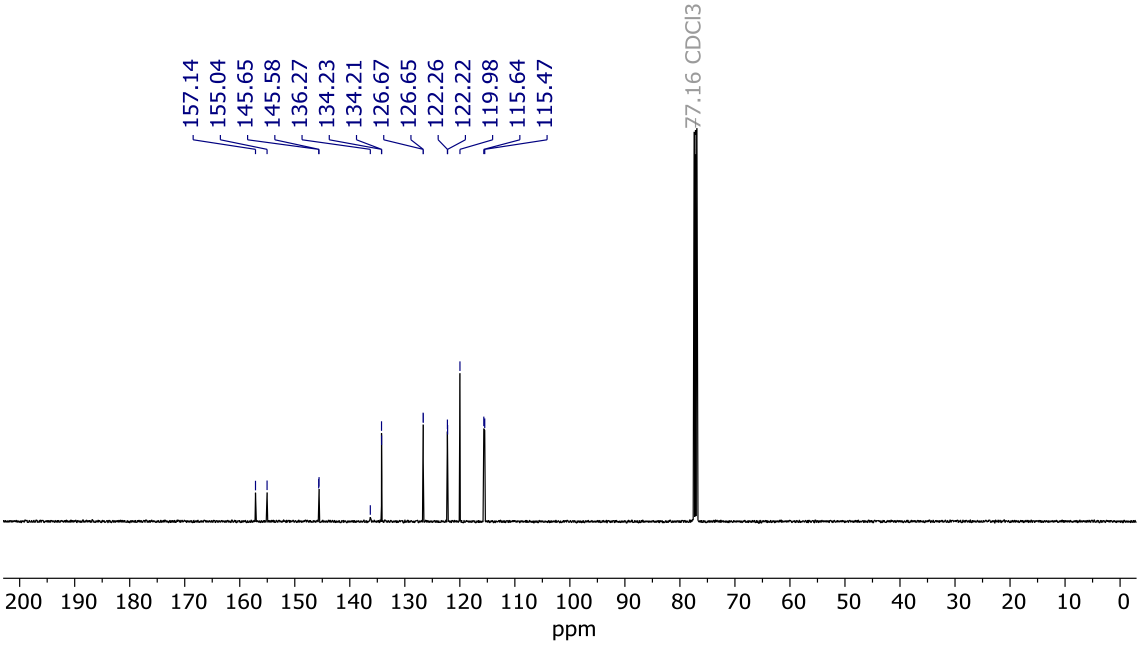

2-((2'-methoxy-4-(4-(2-nitro-5-vinylphenoxy)phenethyl)-[1,1'-biphenyl]-2-yl)oxy) tetrahydropyran (16)

^{1}H NMR (500 MHz, CD_2Cl_2)

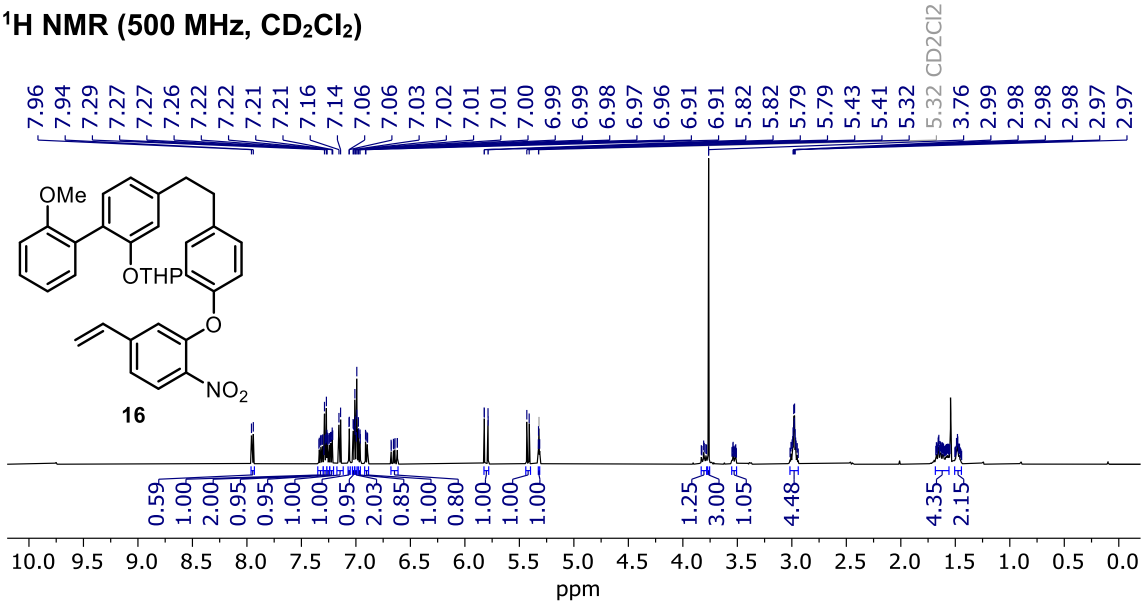

DEPT-135

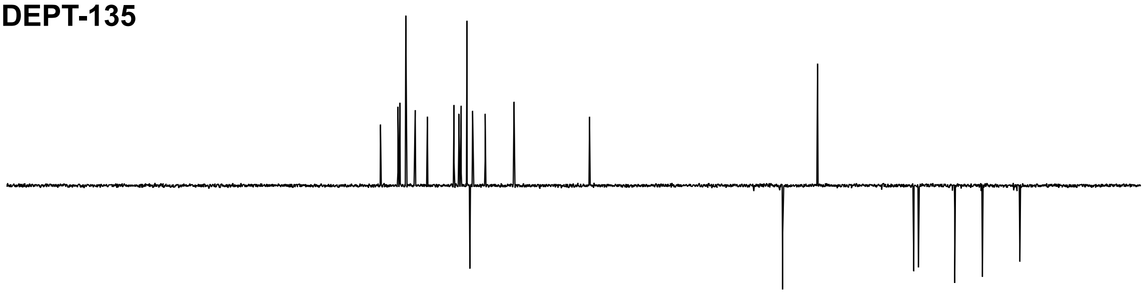

^{13}C NMR (126 MHz, CD_2Cl_2)

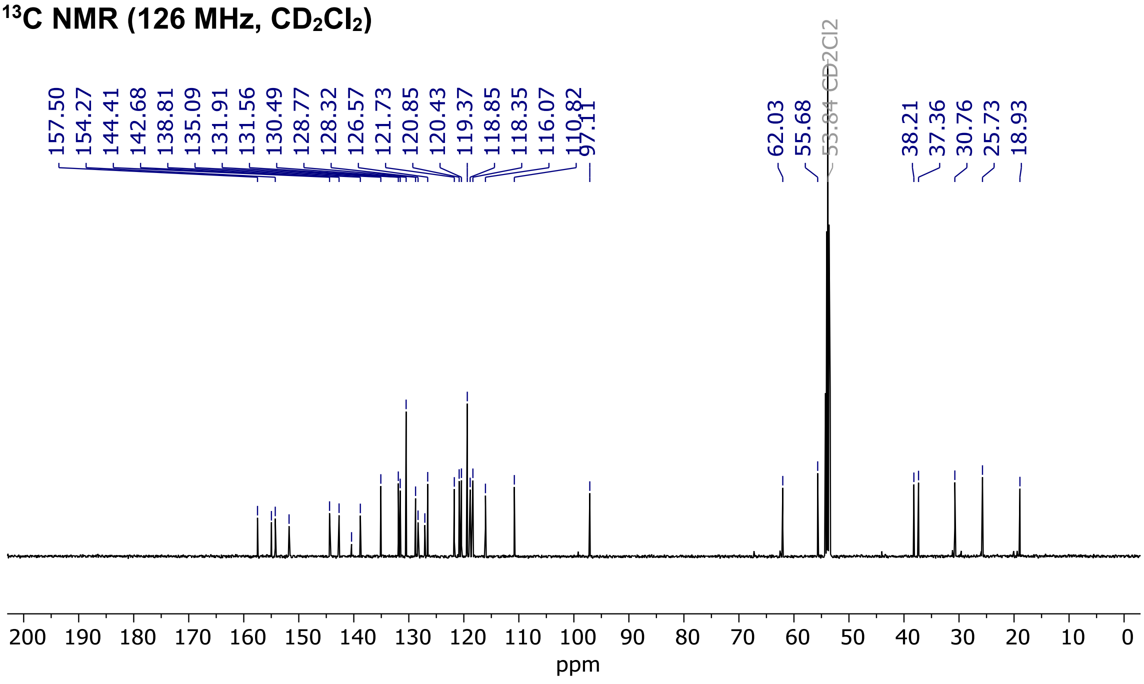

2'-methoxy-4-(4-(2-nitro-5-vinylphenoxy)phenethyl)-[1,1'-biphenyl]-2-ol (17)

^{1}H NMR (500 MHz, $CDCl_3$)

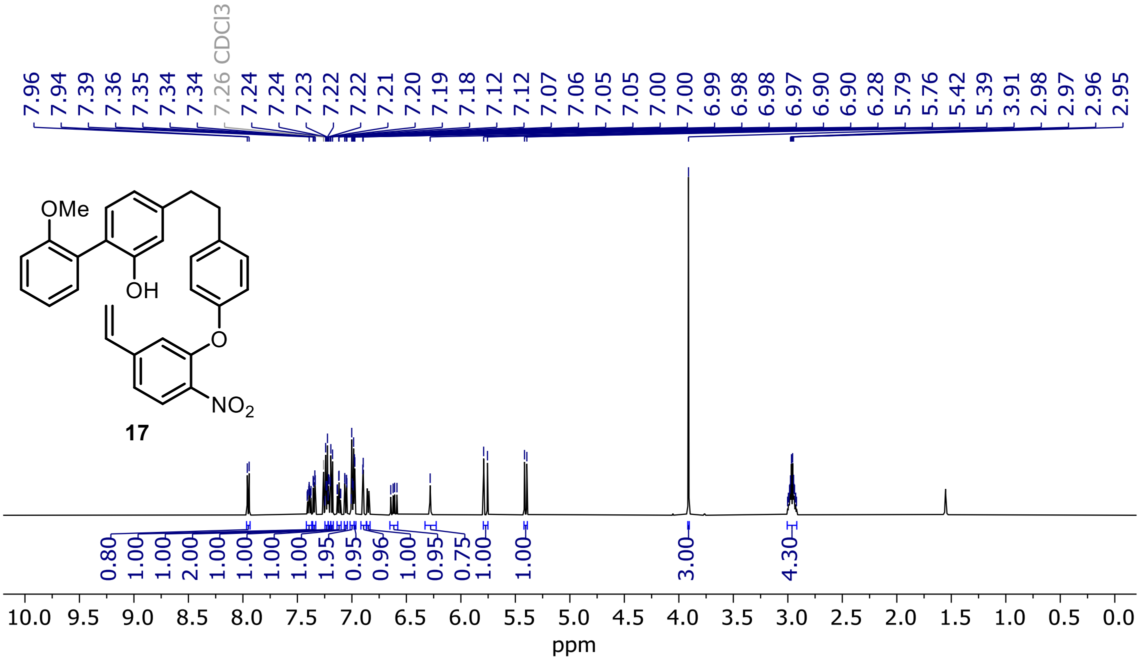

DEPT-135

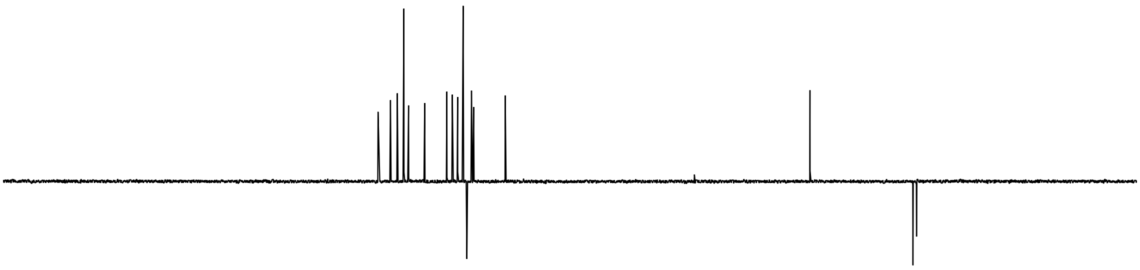

^{13}C NMR (126 MHz, $CDCl_3$)

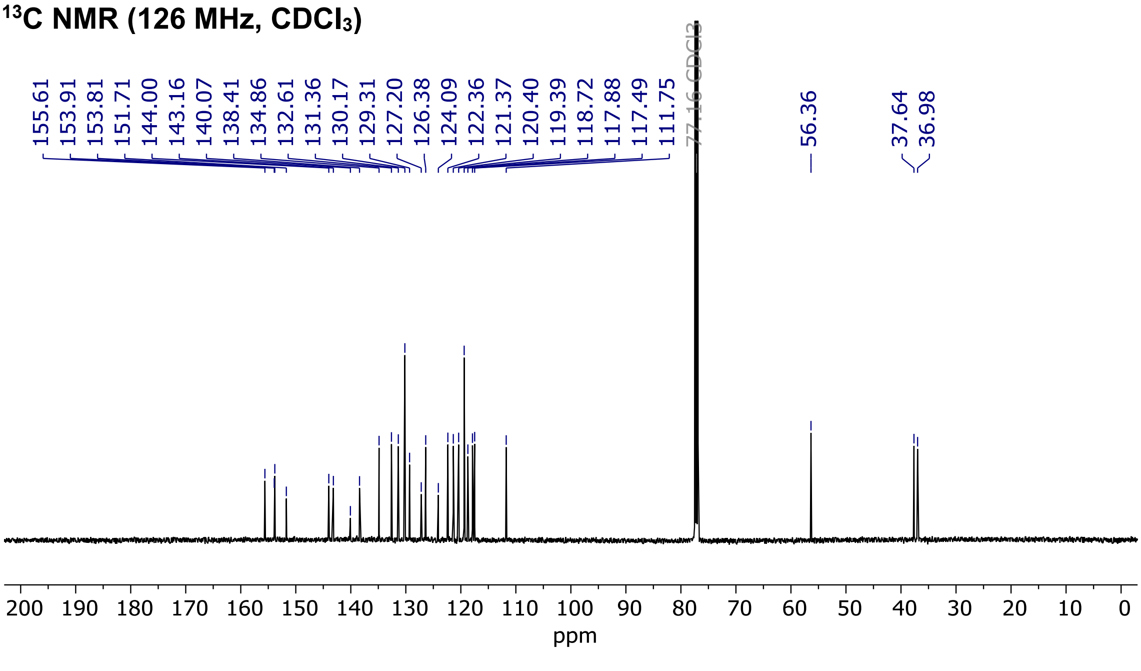

(E)-12-nitro-7,8-dehydroisoriccardin C 3-methyl ether (18)

^{1}H NMR (500 MHz, $CDCl_3$)

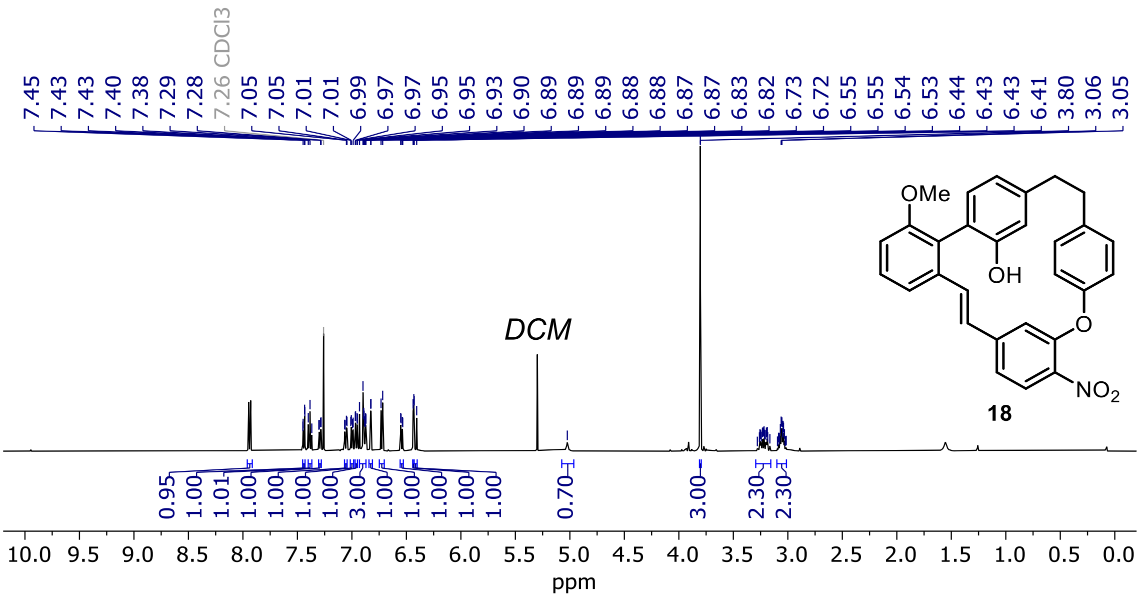

DEPT-135

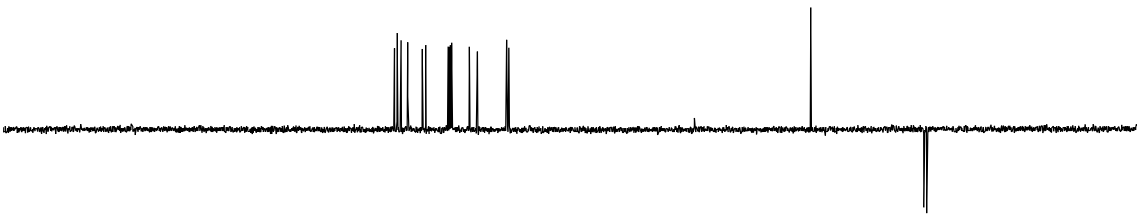

^{13}C NMR (126 MHz, $CDCl_3$)

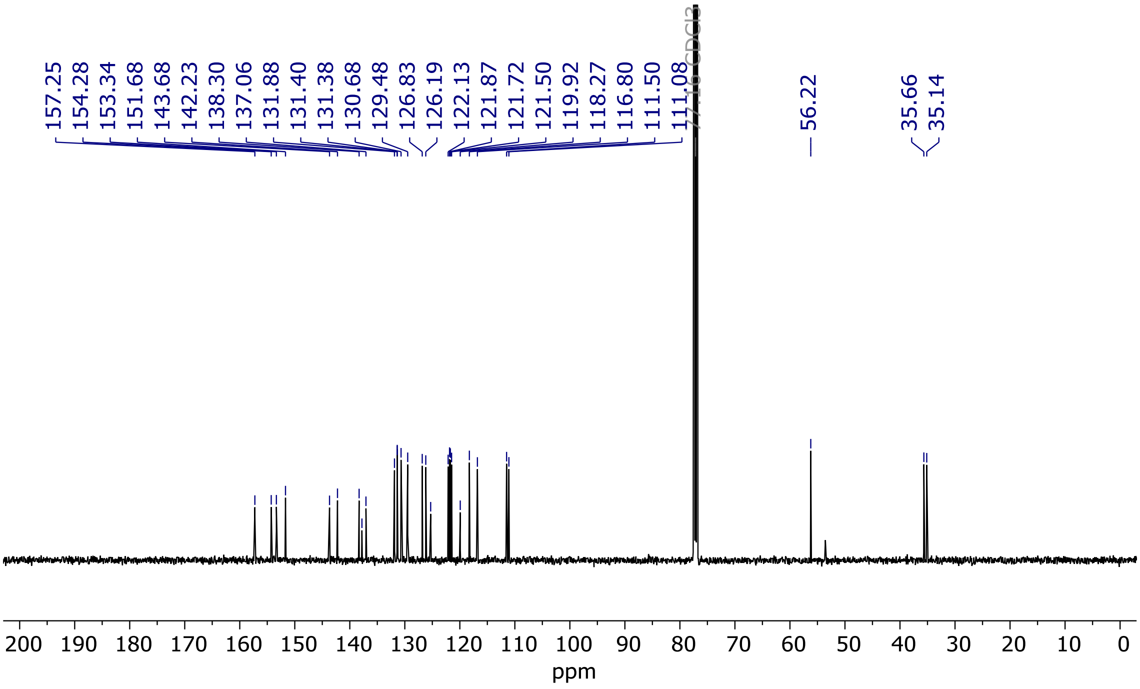

Supplement: Supplementary file 1 [file ol6c00911_si_001.pdf]
